# Overexpression of *GmJAZ5* Enhances Salt Tolerance in Soybean

**DOI:** 10.3390/ijms27177829

**Published:** 2026-09-01

**Authors:** Aixuan Ou, Wenkang Huang, Huan Qiu, Minghong Xie, Tiejun Yang, Yingbin Xue

**Affiliations:** 1 College of Coastal Agricultural Science, Guangdong Ocean University, Zhanjiang 524088, China; oix_gdou2022@163.com (A.O.); 13480168808@163.com (W.H.); qh_gdou2022@163.com (H.Q.); xmh_gdou2025@163.com (M.X.); 2 South China Branch of National Saline-Alkali Tolerant Rice Technology Innovation Center, Zhanjiang 524088, China

**Keywords:** soybean, *GmJAZ5*, salt stress, antioxidant defense

## Abstract

Soil salinization poses a significant challenge to soybean production, yet the regulatory mechanisms underlying root responses to salt stress remain incompletely understood. In this study, we analyzed the expression patterns, subcellular localization, and functions of overexpressed *GmJAZ5* in soybean roots. Under salt stress, *GmJAZ5* expression was consistently downregulated in leaves, whereas roots exhibited initial suppression followed by a recovery phase. The GmJAZ5 protein was localized to the nucleus. *GmJAZ5* overexpression markedly attenuated salt-induced growth inhibition and enhanced the activities of antioxidant enzymes, including superoxide dismutase (SOD), peroxidase (POD), ascorbate peroxidase (APX), and catalase (CAT). This manipulation also reduced malondialdehyde (MDA) accumulation and decreased both Na^+^ content and the Na^+^/K^+^ ratio in leaves. Additionally, it exerted tissue-specific effects on jasmonic acid (JA) and abscisic acid (ABA) levels. These results indicate that overexpression of *GmJAZ5* enhances salt tolerance in soybean by strengthening antioxidant defenses, limiting Na^+^ translocation, and modulating phytohormone homeostasis, highlighting its potential as a candidate gene for breeding salt-tolerant soybean varieties.

## 1. Introduction

Soybean (*Glycine max* (L.) Merr) ranks among the most extensively cultivated and economically significant leguminous crops globally. Its seeds contain approximately 40% protein and 20% lipids, making it an essential source of edible oil, protein-rich animal feed, and dietary protein for human consumption [1]. However, the growth, development, and yield of soybeans are adversely affected by various environmental factors. Notably, soil salinization has emerged as a critical abiotic stressor that limits soybean production. According to data from the Food and Agriculture Organization (FAO) and the United Nations Educational, Scientific and Cultural Organization (UNESCO), around 950 million hectares of salt-affected soils exist worldwide, with this area continuing to grow due to rapid industrialization, improper irrigation practices, and climate change [2]. In China, saline–alkaline lands are prevalent, covering over 30 million hectares and representing more than 10% of the nation’s arable land [3]. Although soybean is considered moderately salt-tolerant, its threshold for soil salinity tolerance is only 5.0 dS/m. Exceeding this threshold significantly inhibits growth and development, ultimately leading to considerable yield losses [4].

Throughout their life cycle, plants are exposed to various environmental stresses, which are broadly classified into biotic and abiotic stresses based on their origin [5]. Among these, abiotic stresses—including salinity, drought, extreme temperatures, and heavy metal toxicity—constitute the primary constraints limiting crop growth, development, and yield formation worldwide, owing to their widespread occurrence and severe impact [6]. Salt stress, in particular, poses a substantial threat to plants: it significantly inhibits seed germination, root elongation, and vegetative growth while severely impairing photosynthesis, dry matter accumulation, and reproductive development, ultimately reducing crop yield and quality; prolonged or intense salt stress can even drive adaptive divergence, profoundly influencing the distribution of crop genetic resources and the direction of breeding programs [7]. The adverse effects of salt stress primarily stem from the excessive accumulation of salts in soil and plant tissues. Under saline–alkaline conditions, Na^+^ and Cl^−^ ions induce osmotic stress, which leads to ionic imbalance, triggering oxidative damage, nutrient disorders, organ senescence, and potentially plant death [8]. From the perspective of stress mechanisms, the adverse effects of soil salinization on plants manifest in two main aspects. The first aspect is osmotic stress. High salinity increases the osmotic potential of the soil solution, which impedes water uptake by roots and causes cellular dehydration. This dehydration ultimately results in leaf wilting and growth arrest [9]. The second aspect is ionic toxicity. The excessive accumulation of Na^+^ and Cl^−^ ions within plant tissues disrupts intracellular ion homeostasis and interferes with various physiological and metabolic processes. These processes include enzyme activities, protein synthesis, and photosynthesis, which can ultimately restrict growth or lead to plant death [10]. Numerous studies have shown that the type and concentration of salts significantly influence plant growth and physiological functions. Higher salt concentrations and prolonged exposure lead to increasingly severe damage to plants [11]. In soybean seedlings, excessive salinity inhibits root growth, markedly reduces the fresh weight of both shoots and roots, decreases plant height, and impairs photosynthetic efficiency. This impairment results in insufficient accumulation of photosynthetic assimilates, ultimately diminishing soybean yield [12].

Over their evolutionary history, plants have developed intricate regulatory networks coordinated by multiple transcription factors to cope with salt stress and other abiotic challenges. By fine-tuning the expression of downstream stress-responsive genes at the transcriptional level, these networks enable plants to withstand adverse conditions [13]. Several transcription factor families, including WRKY, NAC, and MYB, have been demonstrated to play pivotal roles in plant responses to salt and other abiotic stresses. Within the WRKY family, *MbWRKY50* from *Malus baccata* significantly enhances tolerance to low-temperature and drought stress by upregulating antioxidant capacity and promoting reactive oxygen species (ROS) scavenging [14]; *MbWRKY3* from the same species was cloned and functionally validated to enhance drought tolerance in transgenic tobacco, with its mechanism similarly involving reinforcement of the antioxidant system [15]. In the NAC family, overexpression of *FvNAC29* from *Fragaria vesca* enhances tolerance to both salt and low-temperature stress in transgenic *Arabidopsis*, demonstrating broad-spectrum efficacy against multiple abiotic stresses [16]. In the MYB family, overexpression of the grapevine R2R3-MYB transcription factor *VhMYB15* significantly improves *Arabidopsis* tolerance to salt and drought stress, primarily through enhanced osmotic adjustment and activation of antioxidant defense [17]. Collectively, these findings not only underscore the central role of distinct transcription factor families in coordinating plant responses to multiple abiotic stresses but also reveal that, despite structural and mechanistic divergence among upstream transcriptional regulators, the downstream physiological response modules—particularly antioxidant system activation and osmolyte accumulation—exhibit a high degree of conservation across species and stress types. Beyond transcription factors, plant hormone signaling pathways constitute an indispensable component of the abiotic stress response. Among these, jasmonic acid (JA) and its derivatives, as key lipid-derived signaling molecules, play a unique role in mediating plant responses to salt stress.

Jasmonic acid (JA) and its derivatives represent a significant class of plant lipid-derived hormones that play crucial roles in various physiological processes [18,19,20,21]. It has been well established that the JA signaling pathway plays a crucial role in the plant response to salt stress, which markedly induces JA levels, leading to the transcriptional activation of downstream stress-responsive genes [22,23,24,25]. The JAZ (Jasmonate ZIM-domain) proteins act as negative regulators of JA signaling and are crucial for jasmonate signal transduction [26,27,28]. Under basal conditions, JAZ proteins directly bind the transcription factor MYC2 via their Jas motif and, together with the co-repressor TOPLESS recruited through NINJA, repress the transcription of JA-responsive genes [29]. Upon stress, elevated bioactive jasmonoyl–isoleucine (JA-Ile) functions as a molecular glue bridging the Jas motif of JAZ proteins and the F-box protein CORONATINE INSENSITIVE 1 (COI1), triggering 26S proteasome-dependent JAZ degradation to release MYC2 and drive downstream defense gene reprogramming [30,31,32]. In recent years, the roles of *JAZ* family members in modulating plant salt tolerance have garnered considerable attention. Salt stress has been shown to upregulate *JAZ* gene expression in cotton roots [33]. Overexpression of the wild soybean *GsJAZ2* gene significantly enhances the tolerance of transgenic plants to saline–alkali stress [34], while overexpression of *OsJAZ8* in rice affects stress resistance [35], and overexpression of *OsTIFY11a* enhances salt tolerance in rice [36]. In Arabidopsis, salt stress upregulates *AtJAZ1* and *AtJAZ2* expression and mediates root growth inhibition through activation of the JA signaling pathway [37]. Collectively, these studies indicate that JAZ family members play a crucial role in regulating plant salt tolerance by modulating the JA signaling pathway and associated physiological processes, including ion homeostasis, osmotic adjustment, and antioxidant defense. However, the functions of soybean JAZ family members in response to salt stress remain poorly characterized.

The soybean genome encodes more than 20 JAZ family members, which display marked functional divergence. By mining publicly available soybean salt-stress transcriptomic datasets, we identified *GmJAZ5* (*Glyma.06G013700*) as exhibiting a distinct organ-specific expression pattern under salt stress. In this study, using the cultivated soybean variety ‘YC03-3’ as the experimental material, we first analyzed the differential expression patterns and subcellular localization of *GmJAZ5* in leaves and roots under salt stress. Subsequently, a *GmJAZ5* overexpression vector was constructed, and transgenic lines were generated using an Agrobacterium-mediated hairy root transformation system. Transgenic plants were subjected to 0 and 100 mM NaCl treatments, and a comparative analysis was performed across multiple parameters, including growth phenotypes, antioxidant enzyme activities, osmoregulatory substances, Na^+^/K^+^ homeostasis, and JA/ABA hormone levels, to elucidate the physiological mechanisms by which *GmJAZ5* regulates soybean salt tolerance, thereby providing a theoretical foundation and candidate gene resources for breeding salt-tolerant soybean varieties.

## 2. Results

### 2.1. GmJAZ5 Displays Organ-Specific Expression Dynamics in Roots and Leaves Under Salt Stress

**Figure 1:**
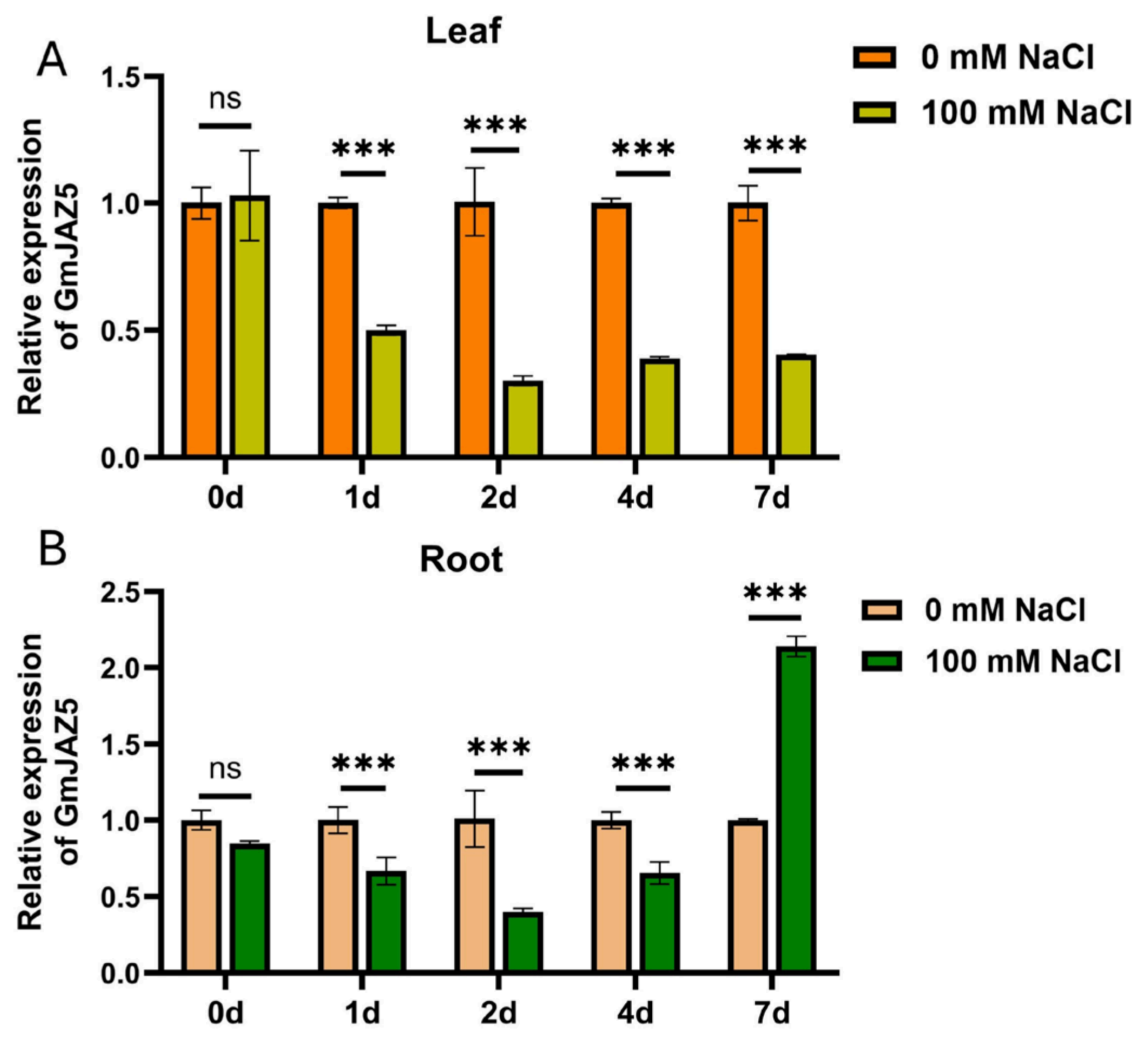
Salt-responsive expression patterns of *GmJAZ5* in soybean leaves and roots. Seedlings of soybean at the fully expanded first trifoliate leaf stage were grown in modified Hoagland’s medium. Leaf and root samples were collected at 0, 1, 2, 4, and 7 days of 100 mM or 0 mM NaCl treatment. Relative expression levels of *GmJAZ5* were determined by qRT-PCR using *GmEF (Glyma.17G186600)* as the internal reference, and fold changes were calculated using the 2^−ΔΔCt^ method. (**A**) Leaves. (**B**) Roots. Data are presented as mean ± SD of three independent biological replicates. Different lowercase letters indicate significant differences among time points (one-way ANOVA followed by Duncan’s multiple range test, ***: *p* < 0.001; ns: no significant difference).

Under salt stress, *GmJAZ5* expression displayed marked tissue-specific divergence between leaves and roots (Figure 1). Throughout the 7-day salt-stress treatment, *GmJAZ5* expression in leaves was persistently suppressed (Figure 1A). In contrast, its expression in roots exhibited a dynamic pattern of early suppression followed by late activation: *GmJAZ5* was significantly downregulated during the early stage (1–4 days); by day 7, its expression had significantly exceeded that of the 0 mM NaCl control, indicative of late-stage induced upregulation (Figure 1B).

### 2.2. GmJAZ5 Protein Is Specifically Localized to the Nucleus

**Figure 2:**
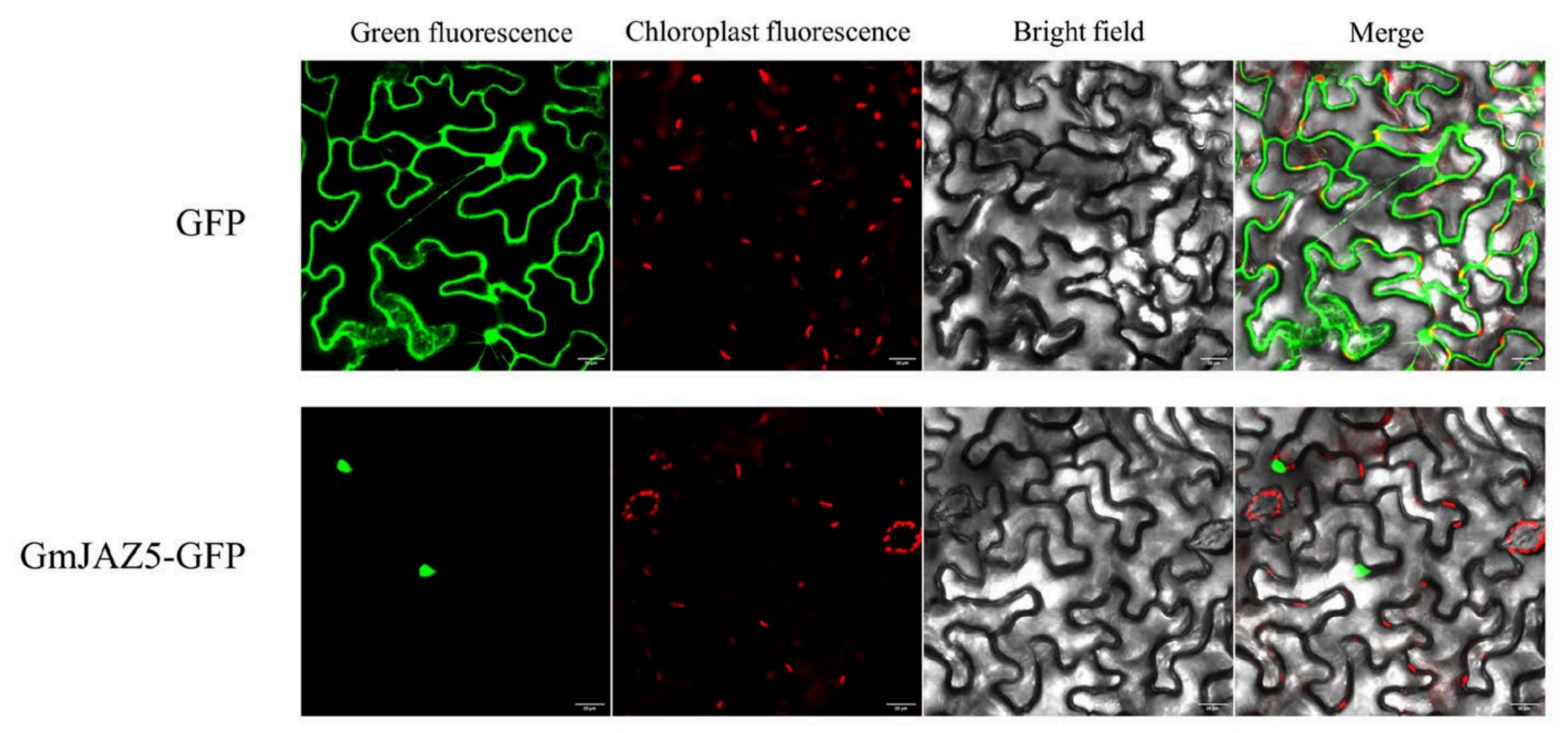
Subcellular localization analysis of the GmJAZ5 protein. The recombinant plasmid pBWA(V)HS-GmJAZ5-eGFP and the empty vector pBWA(V) HS-eGFP (negative control) were individually transformed into Agrobacterium tumefaciens *GV3101* and infiltrated into the abaxial epidermis of leaves of 4- to 6-week-old Nicotiana benthamiana plants. Leaf samples were collected after 48–72 h of incubation at 25 °C and imaged through a laser scanning confocal microscope. GFP, green fluorescence signal (excitation at 488 nm, emission collected at 500–550 nm); chlorophyll, chloroplast autofluorescence (emission collected at 650–750 nm); bright, bright-field image; merged, merged image. Scale bar = 20 μm.

The subcellular localization of *GmJAZ5* was determined by Agrobacterium-mediated transient expression in tobacco leaves. In epidermal cells expressing the *GmJAZ5*-eGFP fusion protein, the green fluorescence signal was predominantly confined to the nucleus and spatially separated from the red autofluorescence of chloroplasts, with negligible fluorescence detected in the cytoplasm. By contrast, in cells expressing the empty vector pBWA(V) HS-eGFP, the eGFP signal was diffusely distributed throughout both the nucleus and the cytoplasm (Figure 2). These results demonstrate that *GmJAZ5* is a nucleus-localized protein.

### 2.3. Overexpression of GmJAZ5 Alleviates Salt-Stress-Induced Growth Inhibition in Soybean

**Figure 3:**
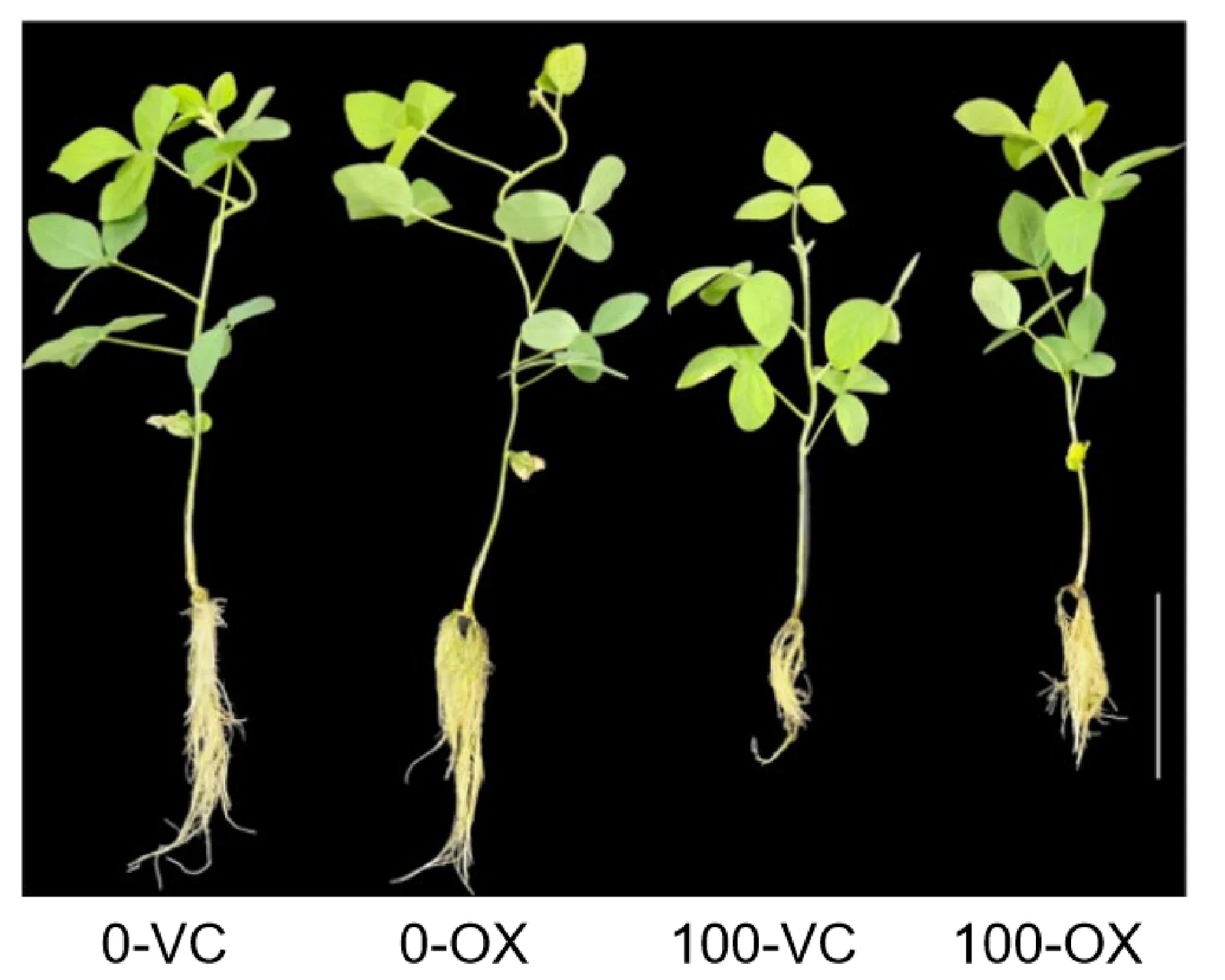
Effects of *GmJAZ5* overexpression on the growth of soybean composite plants. Soybean composite plants, in which the root systems comprised transgenic tissue expressing *GmJAZ5*-pBWA(V)BS or the empty vector pBWA(V)BS, were cultured in modified Hoagland’s medium for 14 days and subsequently treated with medium containing 0 mM NaCl or 100 mM NaCl for 7 days. OX, *GmJAZ5*-overexpressing composite plants (*n* = 15 independent transgenic events, divided into three biological replicates); VC, empty vector control composite plants (*n* = 15). A representative plant from each group is shown. Scale bar = 20 cm.

After 7 days of 100 mM NaCl treatment, plant height, root length, shoot and root fresh weight, and leaf area were significantly reduced in both *GmJAZ5*-overexpressing composite plants (OX) and empty vector control composite plants (VC) compared with the 0 mM NaCl treatment, confirming that salt stress exerted a pronounced inhibitory effect on soybean growth. However, for all measured parameters, the OX line performed significantly better than the VC line; notably, the stress-induced reductions in root length and root fresh weight were markedly attenuated, indicating that overexpression of *GmJAZ5* exerts a stronger protective effect on root growth (Figure 3).

### 2.4. Overexpression of GmJAZ5 Suppresses Excessive ROS Accumulation in Soybean Roots and Leaves Under Salt Stress

**Figure 4:**
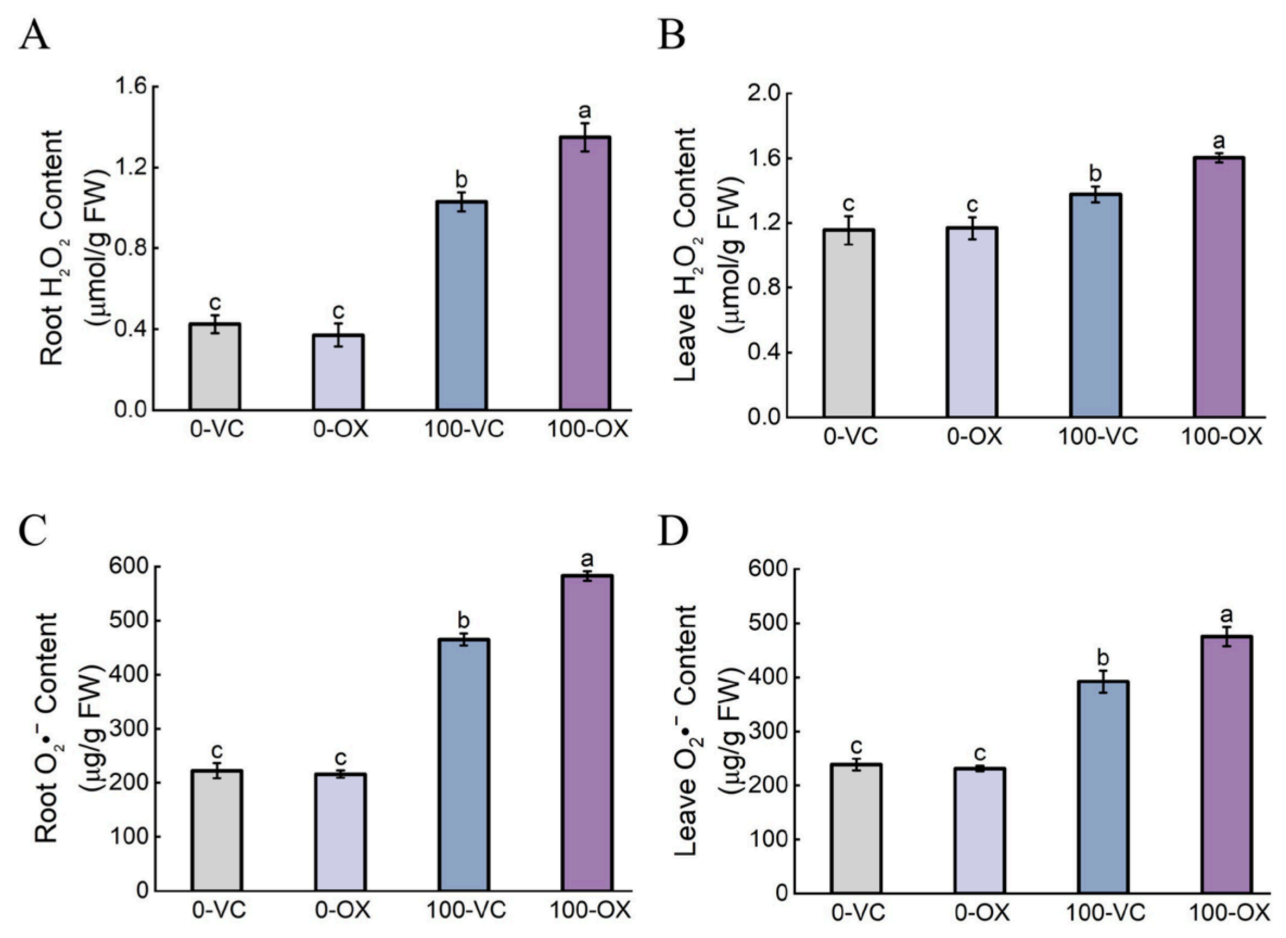
Effects of *GmJAZ5* overexpression on the contents of H_2_O_2_ and O_2_·^−^ in soybean composite plants under salt stress. Soybean composite plants (OX, *GmJAZ5*-overexpressing; VC, empty vector control) were treated with 0 mM or 100 mM NaCl for 7 days. (**A**) H_2_O_2_ content in roots. (**B**) H_2_O_2_ content in leaves. (**C**) O_2_·^−^ generation rate in roots. (**D**) O_2_·^−^ generation rate in leaves. Data are presented as mean ± SD of four independent biological replicates. Different lowercase letters indicate significant differences among treatments (one-way ANOVA followed by Duncan’s multiple range test, *p* < 0.05).

After 7 days of each treatment, H_2_O_2_ content and O_2_·^−^ generation rate were significantly elevated in both roots and leaves of OX and VC lines relative to the 0 mM NaCl control, confirming that salt stress induced marked ROS accumulation. However, the extent of ROS accumulation was significantly lower in the OX line than in the VC line in both organs. Specifically, root H_2_O_2_ content was 23.7% lower (*p* < 0.01) and leaf H_2_O_2_ content was 14.1% lower (*p* < 0.05) in OX compared with VC; similarly, the O_2_·^−^ generation rate in roots was 20.2% lower (*p* < 0.01) and in leaves was 17.5% lower (*p* < 0.05), with the reduction being greater in roots, especially for H_2_O_2_. These results indicate that *GmJAZ5* overexpression effectively restricts salt-stress-induced excessive ROS accumulation and that this alleviating effect is particularly pronounced in roots (Figure 4).

### 2.5. Overexpression of GmJAZ5 Significantly Enhances Antioxidant Defense Capacity in Soybean Under Salt Stress

**Figure 5:**
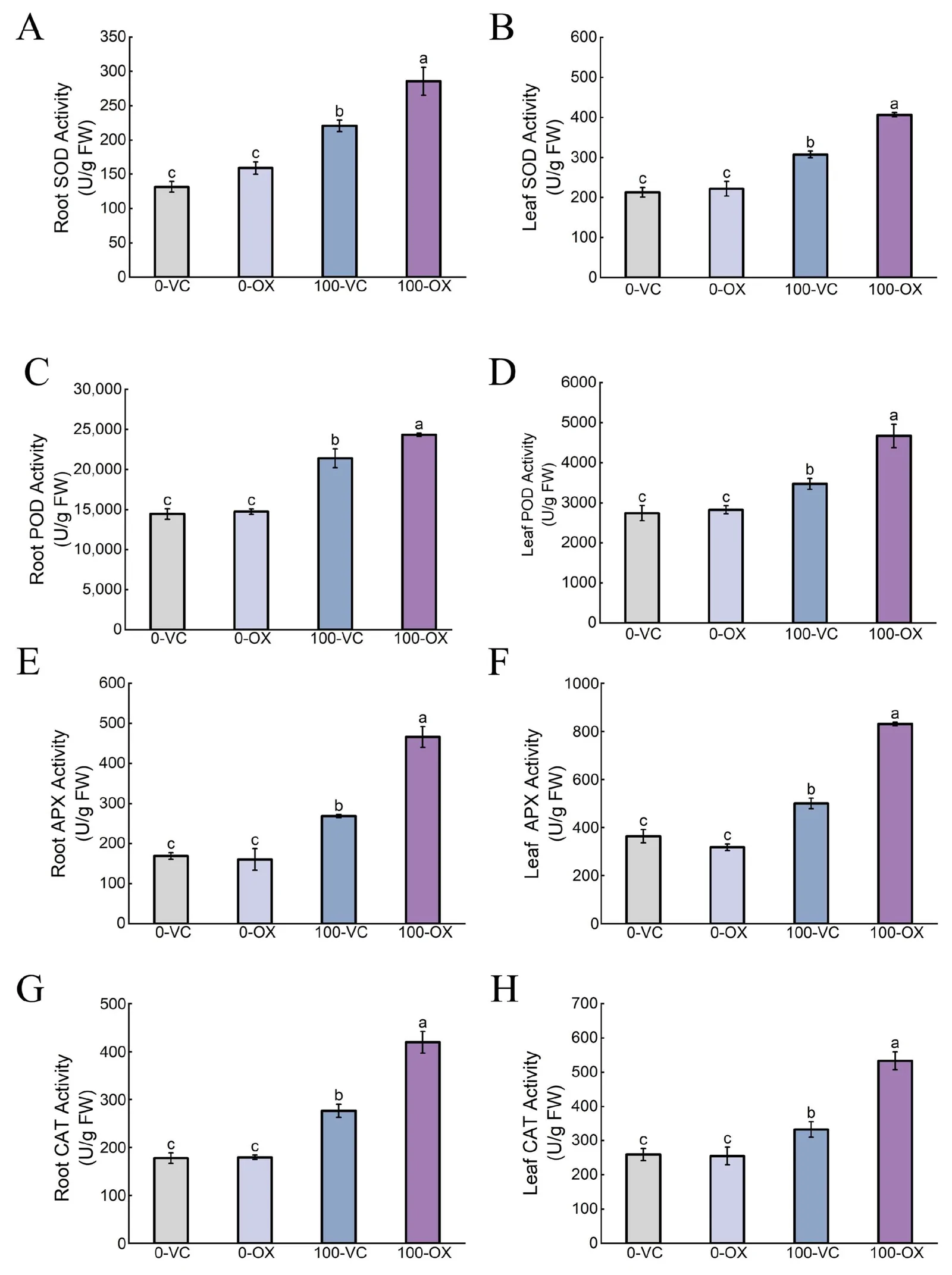
Overexpression of *GmJAZ5* enhances antioxidant enzyme activities in soybean under salt stress. Root and leaf samples were collected from OX (*GmJAZ5*-overexpressing composite plants) and VC (empty vector control composite plants) after 7 days of treatment with 0 mM or 100 mM NaCl. (**A**) Root SOD activity. (**B**) Leaf SOD activity. (**C**) Root POD activity. (**D**) Leaf POD activity. (**E**) Root APX activity. (**F**) Leaf APX activity. (**G**) Root CAT activity. (**H**) Leaf CAT activity. Data are presented as mean ± SD of four independent biological replicates. Different lowercase letters indicate significant differences among treatments (one-way ANOVA followed by Duncan’s multiple range test, *p* < 0.05).

After 7 days of each treatment, the activities of four antioxidant enzymes—superoxide dismutase (SOD), peroxidase (POD), catalase (CAT) and ascorbate peroxidase (APX)—in roots and leaves of both OX and VC lines were significantly higher than those in the 0 mM NaCl control. Moreover, the increase was markedly greater in the OX line than in the VC line, indicating that *GmJAZ5* overexpression enhanced the overall antioxidant defense capacity of soybean (Figure 5). Among these enzymes, the responses of APX and CAT were particularly pronounced: APX activity in OX roots and leaves was approximately 74% and 66% higher than that in VC, respectively, and its salt-induced fold increase (approximately 2.6–2.9-fold) was substantially greater than that in VC (approximately 1.4–1.6-fold). CAT activity exhibited a similar trend, with the salt-induced increase in OX being approximately 2–4 times that observed in VC. In contrast, the increases in SOD and POD activities were relatively modest (approximately 13–34%). These results suggest that *GmJAZ5* may primarily enhance soybean resistance to salt-stress-induced oxidative damage through APX- and CAT-mediated H_2_O_2_ scavenging pathways.

### 2.6. Overexpression of GmJAZ5 Alleviates Membrane Lipid Peroxidation Damage Under Salt Stress

**Figure 6:**
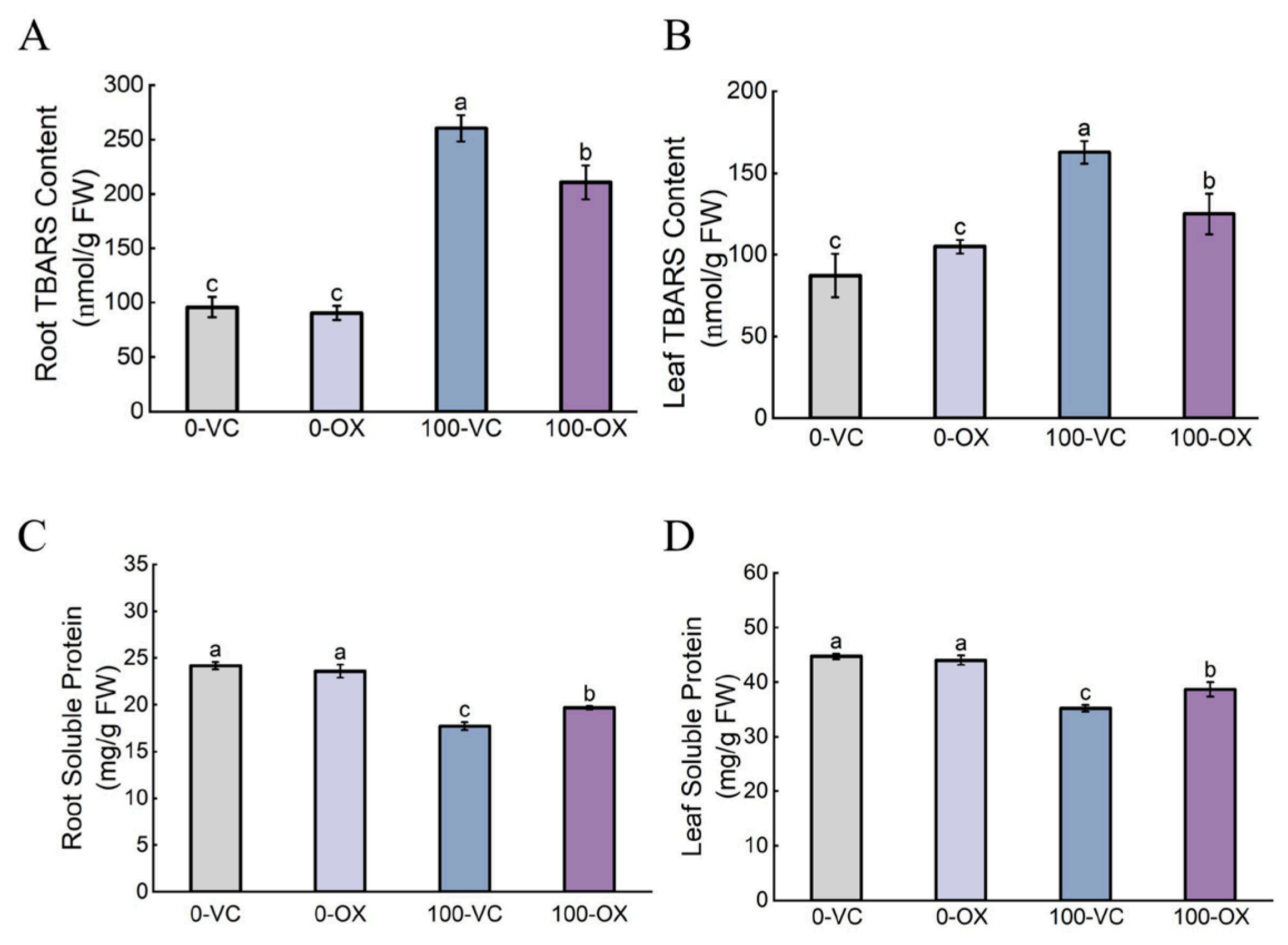
Overexpression of *GmJAZ5* reduces TBARS content and attenuates soluble protein decline under salt stress. After 7 days of treatment with 0 mM or 100 mM NaCl, root and leaf samples were collected for further analysis. OX, *GmJAZ5*-overexpressing composite plants. VC, empty vector control composite plants. (**A**) Root TBARS. (**B**) Leaf TBARS. (**C**) Root soluble protein. (**D**) Leaf soluble protein. Data are presented as mean ± SD of four independent biological replicates. Different lowercase letters indicate significant differences among treatments (one-way ANOVA followed by Duncan’s multiple range test, *p* < 0.05).

Under salt stress, the accumulation of TBARS (thiobarbituric acid-reactive substances, an indirect indicator of membrane lipid peroxidation) in roots and leaves was significantly lower in the OX line than in the VC control, with reductions of approximately 23.3% in roots and 36.4% in leaves. Moreover, the salt-induced increase in leaf TBARS was only approximately 19% in the OX line, far below the approximately 87% increase observed in VC (Figure 6A,B). As a terminal biochemical marker of cell membrane oxidative damage, this marked reduction in TBARS content indicates that the OX line maintained greater membrane structural stability under salt stress. This finding is consistent with the reduced ROS accumulation reported in Section 2.4 and the elevated antioxidant enzyme activities described in Section 2.5, collectively establishing a coherent physiological response chain from ROS scavenging to diminished membrane damage.

In addition, soluble protein content was measured as a supplementary indicator of cellular metabolic activity. Under salt stress, the decline in soluble protein content was significantly attenuated in OX compared with VC, with levels being approximately 11.0% and 10.2% higher in OX roots and leaves, respectively (Figure 6C,D). It should be noted that this relative maintenance of soluble protein content should not be directly interpreted as enhanced osmotic adjustment; rather, it more likely reflects a better-preserved balance between protein synthesis and degradation in OX plants under stress. The specific mechanism requires further investigation through proteomic analyses.

### 2.7. GmJAZ5 Overexpression Reduces Na^+^ Accumulation and Lowers the Na^+^/K^+^ Ratio in Soybean Leaves Under Salt Stress

**Figure 7:**
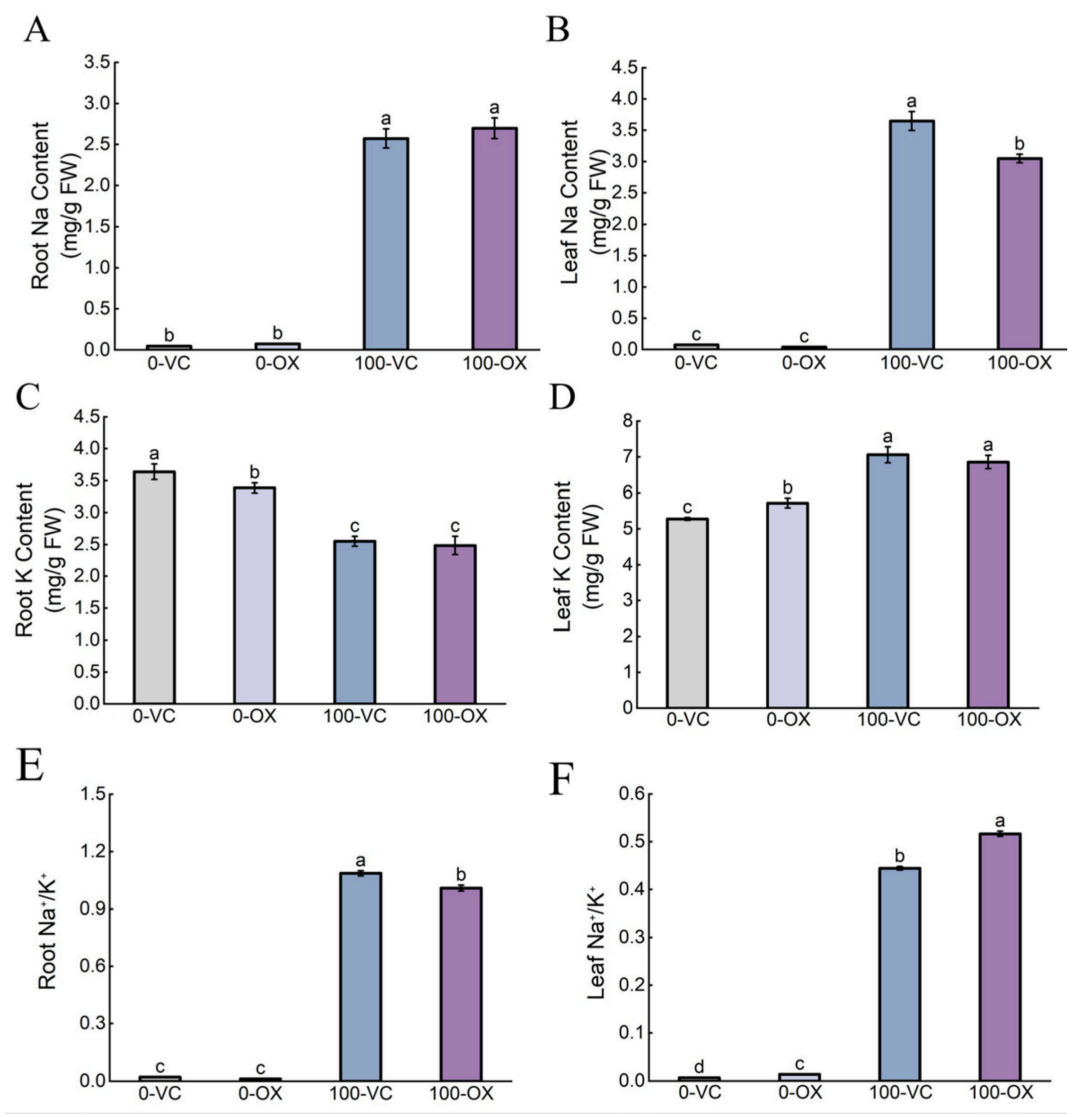
Overexpression of *GmJAZ5* reduces Na^+^ accumulation and Na^+^/K^+^ ratio in soybean roots and leaves under salt stress. OX (*GmJAZ5*-overexpressing composite plants) and VC (empty vector control composite plants) were treated with 0 mM or 100 mM NaCl for 7 days. (**A**) Root Na^+^. (**B**) Leaf Na^+^. (**C**) Root K^+^. (**D**) Leaf K^+^. (**E**) Root Na^+^/K^+^. (**F**) Leaf Na^+^/K^+^. Data are presented as mean ± SD of three independent biological replicates. Different lowercase letters indicate significant differences among treatments (one-way ANOVA followed by Duncan’s multiple range test, *p* < 0.05).

As illustrated in Figure 7, after 7 days of treatment with 100 mM NaCl, the Na^+^ content and Na^+^/K^+^ ratios in both the roots and leaves of all lines were significantly elevated compared to the 0 mM control. In contrast, K^+^ content varied among the lines. Notable differences in ion accumulation patterns were observed between *GmJAZ5*-overexpressing lines (OX) and empty vector control lines (VC). Specifically, both lines exhibited a substantial increase in Na^+^ accumulation following salt-stress treatment. However, the Na^+^ accumulation in the leaves of OX lines was approximately 16.5% lower than that in VC lines. Conversely, Na^+^ content in the roots of OX lines was slightly higher than in VC lines, with only a marginal difference (Figure 7A,B). These findings suggest that the overexpression of *GmJAZ5* effectively limits Na^+^ translocation to the aerial parts, thereby mitigating ionic toxicity in the leaves. Regarding K^+^ content, leaf K^+^ levels in both lines increased following salt-stress treatment, while root K^+^ content decreased by approximately 30% compared to normal conditions. No significant differences in K^+^ content were observed between OX and VC in either leaves or roots. This finding suggests that the overexpression of *GmJAZ5* has limited regulatory effects on K^+^ uptake and maintenance (Figure 7C,D). In contrast, the Na^+^/K^+^ ratio was significantly elevated in all lines under salt stress. Specifically, in leaves, the Na^+^/K^+^ ratio of OX was approximately 14.0% lower than that of VC. This result indicates that OX maintained superior ion homeostasis in leaves. However, the difference in the Na^+^/K^+^ ratio between the two lines in roots was relatively minor.

### 2.8. Overexpression of GmJAZ5 Exerts Organ-Specific Regulation of JA and ABA Accumulation Under Salt Stress

**Figure 8:**
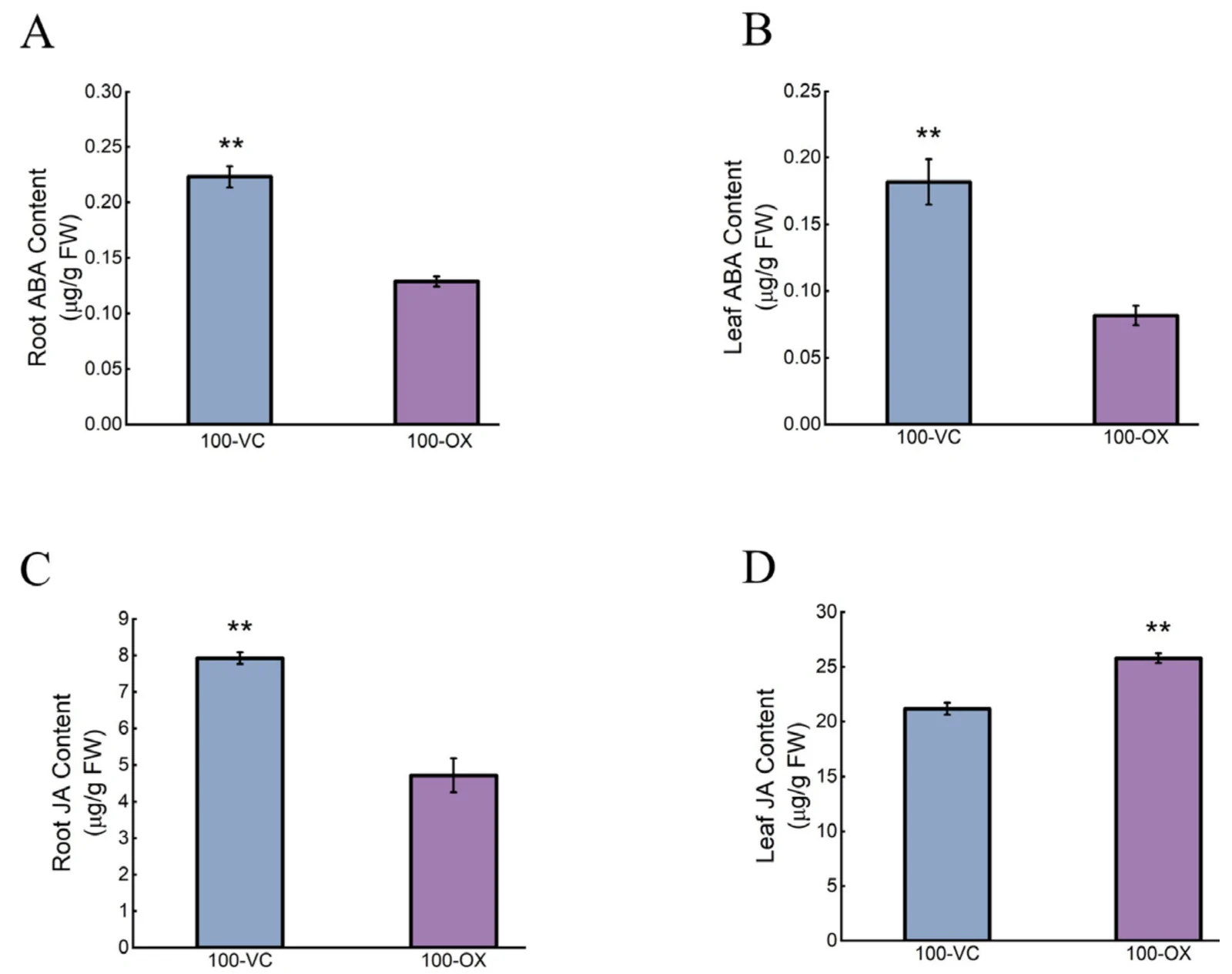
Organ-specific regulation of endogenous hormone contents by *GmJAZ5* overexpression under salt stress. OX (*GmJAZ5*-overexpressing composite plants) and VC (empty vector control composite plants) were treated with 100 mM NaCl for 7 days. Root and leaf samples were collected, and endogenous hormone contents were determined by high-performance liquid chromatography (HPLC). (**A**) Root ABA. (**B**) Leaf ABA. (**C**) Root JA. (**D**) Leaf JA. Data are presented as mean ± SD of three independent biological replicates. Significant differences between OX and VC were analyzed by Student’s *t*-test (** *p* < 0.01).

Under salt stress, JA and ABA accumulation in the OX line exhibited pronounced organ-specific patterns. In leaves, JA content was significantly higher in OX than in VC (increased by approximately 21.8%), whereas ABA content was significantly lower (decreased by approximately 55.0%). In roots, both JA and ABA contents were significantly lower in OX than in VC (decreased by approximately 40.5% and 42.3%, respectively). These findings indicate that *GmJAZ5* participates in the adaptive response of soybean to salt stress through organ-specific hormonal regulation, promoting JA accumulation and suppressing ABA accumulation in leaves while concurrently reducing both JA and ABA levels in roots (Figure 8).

## 3. Discussion

Soil salinization is a major environmental factor limiting soybean production; elucidating the molecular mechanisms underlying soybean salt tolerance and identifying key salt-tolerance genes are central to the development of salt-tolerant varieties. The JA signaling pathway plays a crucial role in the plant response to salt stress; as key negative regulators of this pathway, *JAZ* genes participate in the regulation of plant salt tolerance by modulating the expression of downstream genes. This study found that under salt stress, *GmJAZ5* expression in soybean leaves was persistently suppressed, whereas in roots it exhibited a “short-term suppression–late activation” expression pattern. As the primary organ directly exposed to the saline environment, the root system typically perceives and responds to stress signals more rapidly than aboveground tissues [38]. This dynamic expression pattern is consistent with the classical negative feedback loop in the JA signaling pathway: during early salt stress, the JA-Ile-mediated SCF^COI1^ complex drives the degradation of JAZ proteins to release MYC2 transcriptional activity, and MYC2 subsequently directly activates *JAZ* gene transcription, forming a late-stage feedback compensation mechanism [39]. Therefore, the significant upregulation of *GmJAZ5* at 7 days of root stress may not simply represent a “late-stage stress response,” but rather a molecular marker indicating that JA signaling is approaching a steady state, serving to prevent the sustained inhibition of root growth caused by excessive activation of the JA signaling pathway. In contrast, the continued suppression of *GmJAZ5* expression in leaves suggests that leaves may employ a different strategy for resetting JA signaling or that the JA signaling response in leaves is inherently milder than that in roots. Furthermore, subcellular localization experiments confirmed that the GmJAZ5 protein is specifically localized to the nucleus; this is consistent with the biological function of JAZ proteins as transcriptional regulators, which require interaction with transcription factors such as MYC2 within the nucleus to regulate the expression of downstream genes [40].

This study found that overexpression of *GmJAZ5* under salt stress significantly alleviated growth inhibition in soybean plants, and this protective effect was particularly pronounced in the root system (Figure 3, Table 1). This tissue specificity is closely related to the Agrobacterium-mediated root-specific transgenic system employed in this study. As the root system was the sole transgenic tissue, the significant improvements in root length and fresh weight of the underground parts directly reflect the function of *GmJAZ5* at the cell-autonomous level, whereas improvements in aboveground traits are more likely to be an indirect consequence of systemic effects mediated by the transgenic root system. The root system is the primary organ through which plants absorb water and nutrients; maintaining normal root growth under salt stress is crucial for the salt tolerance of the entire plant [41]. Previous studies have shown that overexpression of the wild soybean *GsJAZ2* can significantly enhance the tolerance of transgenic Arabidopsis to saline–alkali stress [34]. The results of this study are consistent with these findings and further confirm the important role of *JAZ* family members in the regulation of plant salt tolerance.

Salt stress leads to the accumulation of excessive reactive oxygen species (ROS) in plants, causing oxidative damage; plants primarily maintain redox balance through their antioxidant enzyme systems [42]. In this study, overexpression of *GmJAZ5* significantly enhanced the activities of SOD, POD, APX, and CAT in the roots and leaves of soybean under salt stress, and the increase in enzyme activity in the OX line was markedly greater than that in the VC line. Among the four antioxidant enzymes, APX exhibited the most pronounced response; moreover, the increase in its activity was corroborated by a significant reduction in H_2_O_2_ content in OX plants (Figure 4), suggesting that the ascorbic acid–glutathione (AsA-GSH) cycle may be the preferred pathway through which *GmJAZ5* regulates antioxidant defense. APX exhibits a higher affinity for H_2_O_2_ than CAT and is capable of finely regulating the redox balance in the cytoplasm and chloroplasts under low-concentration H_2_O_2_ conditions [43]; consequently, its preferential activation in OX is particularly important for maintaining cellular redox homeostasis under prolonged salt stress. A similar phenomenon, whereby JA signaling preferentially activates the APX/AsA-GSH pathway, has also been reported in various crops such as rice [44] and has been systematically summarized in a recent review [21], suggesting that this may be a conserved mechanism by which the JA-JAZ module regulates plant antioxidant defense. This finding is consistent with reports that the JA signaling pathway can activate the antioxidant enzyme system [45]. It should be noted that this study did not directly measure the transcriptional levels of antioxidant enzyme genes; therefore, whether *GmJAZ5* directly targets genes such as *GmAPX* and *GmSOD* via transcriptional regulation remains speculative. As a classic JAZ inhibitor, GmJAZ5 is more likely to indirectly influence the transcriptional response at the G-box elements on the promoters of downstream antioxidant genes by regulating the activity of MYC2-type transcription factors [40]. This inference requires further validation through transcriptomic analysis, promoter binding assays (such as ChIP-qPCR), and verification of the MYC2–GmJAZ5 interaction. Furthermore, this study found that overexpression of *GmJAZ5* significantly reduced MDA accumulation under salt stress, which is closely associated with increased antioxidant enzyme activity. Since MDA is the end product of the peroxidation of polyunsaturated fatty acids initiated by ·OH and O_2_·^−^ [46], the reduction in O_2_·^−^ levels in OX plants and the decrease in MDA accumulation are highly consistent from a biochemical perspective, jointly supporting the integrated conclusion that “GmJAZ5 primarily maintains membrane system integrity by limiting upstream ROS production and accelerating their clearance”. Soluble proteins are important osmoregulatory substances in plants; their accumulation under salt stress helps cells maintain osmotic pressure and water balance [47]. In this study, the soluble protein content of both lines decreased under salt stress, but the decrease in the OX line was significantly smaller than that in the VC line. This “protein protection” effect is unlikely to stem solely from enhanced de novo protein synthesis; rather, it is more likely an indirect consequence of reduced protein oxidative damage and proteolytic hydrolysis resulting from the lower ROS burden in OX plants. Similar phenomena have been reported in other plants overexpressing stress-tolerant *JAZ* genes [34], further supporting the view that improved redox homeostasis can indirectly stabilize the cellular proteome under stress conditions.

From a broader transcriptional regulatory perspective, the mechanism of *GmJAZ5* elucidated in this study—alleviating salt-stress damage by enhancing antioxidant enzyme activity and reducing ROS accumulation—exhibits striking convergence with the functional mechanisms of various transcription factors recently implicated in abiotic stress responses. For instance, *MbWRKY50* from *Malus baccata* confers tolerance to low-temperature and drought stress by upregulating antioxidant capacity and promoting ROS scavenging [14], a regulatory pattern closely aligned with the mechanism described herein, whereby *GmJAZ5* activates SOD, POD, APX, and CAT while reducing H_2_O_2_ and O_2_·^−^ accumulation; the homologous *MbWRKY3* similarly enhances drought tolerance in transgenic tobacco by boosting antioxidant enzyme activity and osmotic adjustment capacity [15]. Furthermore, grapevine *VhMYB15* significantly enhances *Arabidopsis* tolerance to salt and drought stress by strengthening osmotic adjustment and antioxidant defense [17], while overexpression of *FvNAC29* from *Fragaria vesca* concurrently enhances salt tolerance and cold tolerance in transgenic plants, further corroborating the central role of the antioxidant system in stress responses mediated by diverse transcription factors [16]. Collectively, evidence from distinct transcription factor families—including WRKY, NAC, MYB, and the JAZ family investigated here—points to a fundamental principle: despite marked family-specific differences in upstream transcriptional regulatory components, the key downstream physiological response modules, particularly antioxidant system activation and the coordination of Na^+^/K^+^ homeostasis with osmolyte accumulation, are highly conserved across species and abiotic stress types. The multi-level physiological effects exhibited by *GmJAZ5* in this study represent a concrete manifestation of this conserved response network in the context of soybean salt-tolerance regulation. This raises a compelling question: does *GmJAZ5*, through interactions with downstream transcription factors such as MYC2, indirectly modulate the transcriptional activity of promoter regions of genes associated with antioxidant defense and ion transport, thereby forming functional synergy or cross-regulation with the regulatory networks of WRKY, NAC, and MYB transcription factors? Addressing this question will not only advance the elucidation of the complete molecular mechanism of the JA–JAZ module in soybean abiotic stress responses but also provide new candidate targets for the molecular breeding of salt-tolerant soybean varieties.

Maintaining intracellular ion homeostasis is one of the key mechanisms underlying plant salt tolerance [48]. This study found that overexpression of *GmJAZ5* significantly reduced Na^+^ accumulation and the Na^+^/K^+^ ratio in soybean leaves under salt stress but had no significant effect on K^+^ content. At the same time, Na^+^ content in OX roots was not significantly lower than that in VC roots; this distribution pattern of “Na^+^ retention in roots versus Na^+^ exclusion from leaves” is phenotypically highly similar to the classic mechanism of xylem Na^+^ reuptake mediated by SOS1-type plasma membrane Na^+^/H^+^ antiporters and HKT1-type Na^+^ transporters [4]. Similarly, overexpression of *TaNHX2* promotes Na^+^ transport into vacuoles, reducing cytoplasmic Na^+^ concentrations and thereby alleviating ion toxicity [49]. However, the current data do not allow a direct determination of which class of Na^+^ transporters GmJAZ5 acts upon. Previous studies have shown that JA signaling can regulate crosstalk between various plant hormone signaling pathways and Na^+^ transport pathways [50], providing a plausible but as yet unverified molecular basis for the involvement of the JA-JAZ module in the regulation of ion homeostasis. Therefore, future work should involve systematically analyzing the differential expression of key Na^+^ transport genes—such as *GmSOS1*, *GmHKT1* and *GmNHX1*—in the root systems of OX and VC plants, combined with radioisotope tracing experiments to elucidate the specific molecular pathways through which *GmJAZ5* regulates Na^+^ distribution.

JA and ABA are two important hormones involved in the plant response to salt stress [51]. This study found that the JA levels regulated by *GmJAZ5* overexpression exhibit organ specificity, which are decreased in roots but increased in leaves (Figure 8). It is worth noting that, as a negative regulator of JA signaling, overexpression of JAZ proteins should, in theory, inhibit the JA signaling pathway; however, in this study, JA levels in OX leaves were actually elevated. The JA signaling pathway involves a classical negative feedback loop: once MYC2 is released from JAZ inhibition, it can directly activate the transcription of JAZ genes and key genes involved in JA synthesis (LOX, AOS, AOC, and OPR3), thereby enhancing JA biosynthesis [38]. As the root system serves as the initial organ to perceive salt stress, the strong local induction of GmJAZ5 expression (Figure 1B) may transiently enhance this feedback loop, thereby paradoxically increasing the local rate of JA biosynthesis in the root system. However, due to the strong inhibition of JA signaling by the overexpressed GmJAZ5 protein itself, the steady-state JA level ultimately shows a net decrease in the OX root system. In addition, JA and its precursor JA-Ile can be transported over long distances via the phloem; JAZ-mediated signaling inhibition in source organs may alter the systemic distribution of JA [52]. Consequently, OX plants may allocate a greater proportion of JA metabolic flux to the root system, whilst the steady-state accumulation of JA in the leaves following transport actually increases. Moreover, differences in the organ-specific expression of JA-inactivating enzymes (such as JOX and ST2a) between OX and VC plants may also contribute to this asymmetric distribution [53]. The reduction in ABA content in both roots and leaves may be related to the regulation of the growth–defense balance mediated by *GmJAZ5*; lower ABA levels may help to mitigate the inhibitory effect of ABA on growth, enabling the plants to maintain a certain capacity for growth under salt stress [54].

In this study, we systematically analyzed the regulatory role of GmJAZ5 in the soybean response to salt stress across growth phenotypes, antioxidant defense, membrane lipid peroxidation, ion homeostasis, and endogenous hormone levels. The results indicate that *GmJAZ5* overexpression significantly alleviated growth inhibition in soybean plants under 100 mM NaCl stress. In terms of oxidative stress, the accumulation of H_2_O_2_ and O_2_·^−^ in the roots and leaves of OX plants was significantly reduced, while the activities of the four antioxidant enzymes—SOD, POD, APX, and CAT—were significantly enhanced, indicating that *GmJAZ5* may activate antioxidant defense pathways via the JAZ-MYC downstream transcriptional regulatory network. In terms of ion homeostasis, the accumulation of Na^+^ and the Na^+^/K^+^ ratio in the leaves of OX plants were significantly reduced, whereas no significant changes were observed in the roots, exhibiting a distribution pattern characterized by “Na^+^ retention in the roots and Na^+^ exclusion from the leaves,” suggesting that *GmJAZ5* may be involved in regulating the long-distance transport and inter-organ redistribution of Na^+^. In terms of hormonal regulation, JA levels in the leaves of OX plants were elevated, whereas ABA levels were reduced; conversely, both JA and ABA levels in the roots were simultaneously reduced, demonstrating a distinct organ-specific regulatory pattern.

It should be noted that in the Agrobacterium-mediated composite plant system used in this study, only the root system was transgenic, whilst the aboveground leaves remained wild-type; therefore, the leaf-related phenotypes may partly stem from systemic signals mediated by the transgenic root system, rather than the cell-autonomous function of *GmJAZ5* in the leaves. This point requires further verification using whole-plant stable transformation lines. Furthermore, this study did not directly analyze the expression levels of antioxidant enzyme-encoding genes such as *GmSOD1*, *GmAPX2*, and *GmCAT1*, nor those of Na^+^ transporters such as *GmSOS1*, *GmHKT1* and *GmNHX1*. Consequently, the specific regulatory mechanisms of GmJAZ5 on these targets remain speculative and require systematic validation through molecular biological approaches such as transcriptomics, protein interaction assays, and ChIP-seq/DAP-seq. Despite these limitations, this study is the first to reveal, at the physiological level, the functional role of *GmJAZ5* in positively regulating salt tolerance in soybean. It provides experimental evidence for an in-depth analysis of the molecular mechanisms by which the JA signaling pathway participates in soybean’s response to abiotic stress and serves as a reference resource for the identification of candidate genes in the development of salt-tolerant soybean germplasm.

## 4. Materials and Methods

### 4.1. Plant Materials and Treatments

This experiment was conducted at the College of Coastal Agricultural Sciences, Guangdong Ocean University, utilizing the soybean cultivar ‘YC03-3’. Healthy and plump soybean seeds were surface-sterilized in a 0.1% sodium hypochlorite (NaClO) solution for 5 min, followed by five rinses with distilled water. After rinsing, the seeds were sown in quartz sand for germination. Five days later, seedlings exhibiting uniform growth were selected and transferred to a nutrient solution containing 1500 μM KNO_3_, 1200 μM Ca(NO_3_)_2_·4H_2_O, 500 μM KH_2_PO_4_, 500 μM MgSO_4_ ·7H_2_O, 400 μM NH_4_NO_3_, 300 μM K_2_SO_4_, 300 μM (NH_4_)_2_SO_4_, 40 μM Fe-EDTA(Na), 25 μM MgCl_2_, 2.5 μM Na_2_B_4_O_7_·10H_2_O, 1.5 μM ZnSO_4_·7H_2_O, 0.5 μM CuSO_4_·5H_2_O, and 0.16 μM (NH_4_)_6_Mo_7_O_24_·4H_2_O, for hydroponic cultivation in a growth chamber maintained at 26 °C, under a 16 h light and 8 h dark photoperiod. The nutrient solution was renewed every 7 days. Salt stress was applied once the first trifoliate leaves were fully expanded. Two NaCl concentrations, 0 and 100 mmol·L^−1^, were administered, and leaf and root samples were collected at 0, 1, 2, 4, and 7 days following the initiation of salt-stress treatment, with three biological replicates for each tissue type. The nutrient solution utilized was a modified nutrient solution. All chemicals employed were of analytical grade and procured from Solarbio (Beijing, China). For subsequent experimental analyses, samples collected for physiological measurements were immediately frozen in liquid nitrogen and stored at −80 °C.

*Nicotiana benthamiana* plants were cultivated in plastic pots filled with a 1:1 (*v*/*v*) mixture of vermiculite and nutrient soil within a controlled environment chamber. The chamber maintained a photoperiod of 16 h light and 8 h dark, with a light intensity of 300 μmol·m^−2^·s^−1^, a temperature of 25 ± 2 °C, and relative humidity ranging from 60% to 70%. For transient transformation experiments, plants aged 4 to 6 weeks were utilized.

### 4.2. RNA Extraction and Quantitative Real-Time PCR Analysis

Total RNA was extracted from soybean roots and leaves treated with 0 and 100 mM NaCl for 7 days using the MolPure^®^ Plant RNA Kit (Yeasen, Shanghai, China). To eliminate genomic DNA contamination, DNase I (Yeasen, Shanghai, China) was employed. RNA concentration and purity were determined using a NanoDrop 2000 microspectrophotometer (Thermo Fisher Scientific, Waltham, MA, USA), and integrity was verified by 1% denaturing agarose gel electrophoresis. Following this, first-strand cDNA was synthesized using the Hifair^®^ II 1st Strand cDNA Synthesis Kit (Yeasen, Shanghai, China). Quantitative real-time PCR (qRT-PCR) was conducted on a Bio-Rad CFX Maestro real-time PCR system (Bio-Rad, Carlsbad, CA, USA) [55]. The 20 µL qRT-PCR reaction mixture consisted of the following components: 10 µL Hieff UNICON^®^ Universal Blue qPCR SYBR Green Master Mix (2×, final concentration 1×, Yeasen, Shanghai, China), 0.5 µL forward primer (10 μM, final concentration 0.25 μM), 0.5 µL reverse primer (10 μM, final concentration 0.25 μM), 1 µL cDNA template (approximately 50 ng) and 8 µL nuclease-free water. The qRT-PCR protocol was as follows: pre-denaturation at 95 °C for 30 s; then 40 cycles of denaturation at 95 °C for 5 s and annealing/extension at 58 °C for 60 s, with a final extension at 72 °C for 30 s. *GmEF (Glyma.17G186600)* was selected as the internal reference gene. The full sequences of all qRT-PCR primers are provided in Supplementary Table S1. Relative gene expression levels were calculated using the 2^−ΔΔCt^ method [56]. Each sample comprised three biological replicates, with three technical replicates per biological replicate. Raw Ct values are in Supplementary Table S2.

### 4.3. Cloning of the GmJAZ5 Gene and Construction of an Expression Vector

Specific amplification primers with homologous arms were designed based on the CDS sequence (504 bp) of *GmJAZ5 (Glyma.06G013700)* from the soybean genome database (Phytozome v13, Wm82.a4.v1). The 5′ ends of both primers contained a 20 bp homologous arm sequence matching the linearized end of the target vector. The forward primer *GmJAZ5-F* was 5′-TACAATTACGGATCCACGCGATGGAGTTGTTTCCCTCAATCAAGGCAC-3′, and the reverse primer *GmJAZ5-R* was 5′-TCCTCGCCCTTGCTCACCATAGATCCTCCTCCAGATCCTCCTC-3′. Using cDNA from root tissue of YC03-3 treated with salt stress for 7 days as the template, PCR amplification was performed with 2× Biorun Pfu High-Fidelity DNA Polymerase (Biorun, Wuhan, China). The 50 μL reaction mixture comprised 20 μL of nuclease-free water, 25 μL of 2× Biorun Pfu PCR Mix, 2 μL each of the forward and reverse primers (100 μM), and 1 μL of cDNA template. The amplification program was as follows: pre-denaturation at 94 °C for 5 min; 30 cycles of denaturation at 94 °C for 30 s, annealing at 50 °C for 45 s, and extension at 72 °C for 30 s; followed by a final extension at 72 °C for 10 min. The amplification products were separated by 1.5% agarose gel electrophoresis; the 504 bp target band was excised and purified using a DNA gel recovery kit (Axygen, Union City, CA, USA) and finally dissolved in 40 μL of nuclease-free water for later use.

Vector linearization was performed using a dual-restriction enzyme system comprising MluI and BsaI. The 20 μL reaction mixture contained 12 μL of nuclease-free water, 2 μL of 10× buffer, 1 μL of MluI, 1 μL of BsaI, and 4 μL of either the subcellular localization vector pBWA(V)HS-eGFP or the overexpression vector pBWA(V)BS (Wuhan Boyuan Biotechnology, Wuhan, China). Both vectors carry a kanamycin prokaryotic selection marker; the pBWA(V)HS-eGFP backbone also contains a hygromycin eukaryotic selection marker, while the pBWA(V)BS backbone carries the bar herbicide-resistance gene for the selection of positive plant transformants. Digestion was carried out at 37 °C for 60 min. The digestion products were purified using a PCR purification kit prior to subsequent recombination reactions.

Recombination and ligation were performed using the BioRun Seamless Cloning Kit (BioRun, Wuhan, China) following the principle of Gibson assembly [57]. A 20 μL reaction mixture comprising 2 μL of nuclease-free water, 10 μL of 2× EasyClone Mix, approximately 100 ng of the purified *GmJAZ5* PCR product, and approximately 50 ng of the linearized vector was incubated at 37 °C for 30 min to complete homologous recombination. Then, 10 μL of the ligation product was used to transform Escherichia coli DH5α competent cells, which were then spread onto LB solid medium containing kanamycin (50 μg·mL^−1^). Plates were incubated inverted at 37 °C for 12 h, followed by colony PCR identification. The identification primers *TMV-F* (5′-TTGAGGCAACACAATTACCAAC-3′) and *NOSseq-R* (5′-CAAGACCGGCAACAGGATTCAATC-3′) amplified the expected target band of approximately 1417 bp. A single positive colony was inoculated into 5 mL of LB liquid medium containing kanamycin, incubated overnight at 37 °C with shaking, and 100 μL of the culture was sent to Shenggong Biotechnology (Shanghai, China) for verification by Sanger bidirectional sequencing. After sequence alignment confirmed that the sequences were entirely correct, the recombinant plasmids were named pBWA(V)HS-GmJAZ5-eGFP (for subcellular localization) and pBWA(V)BS-GmJAZ5 (for genetic transformation of soybean root hairs). The plasmids were subsequently extracted and stored at –20 °C for future use.

### 4.4. Subcellular Localization

The pBWA(V)HS-GmJAZ5-eGFP fusion plasmid and the empty vector pBWA(V)HS-eGFP were introduced separately into Agrobacterium tumefaciens strain *GV3101* using the freeze–thaw method. Positive colonies were cultured overnight at 28 °C in YEP liquid medium containing kanamycin (50 μg·mL^−1^) and rifampicin (50 μg·mL^−1^). Subsequently, the bacterial cells were resuspended in infiltration buffer (10 mM MgCl_2_, 10 mM MES pH 5.6, 100 μM acetosyringone) to achieve an OD_600_ of 1.0. The suspension was then incubated at room temperature in the dark for 3 h. Using a needleless syringe, the bacterial suspension was infiltrated into the abaxial epidermis of 4- to 6-week-old *N. benthamiana* leaves. The infiltrated plants were maintained at 25 °C under a photoperiod of 16 h light and 8 h dark for 48 to 72 h. Epidermal peels from the infiltrated leaf regions were prepared and examined with a Leica TCS SP8 confocal laser scanning microscope. eGFP fluorescence was excited at 488 nm, with emission collected between 500 and 550 nm; chloroplast autofluorescence was collected between 650 and 750 nm. Leaves expressing the empty vector served as negative controls [58]. For each treatment, five leaves were examined independently, and 10 fields of view were randomly selected from each leaf.

### 4.5. Transformation of Soybean Root Hair Cells and Identification of Transgenic Plants

The *Agrobacterium*-mediated transformation protocol for soybean root hairs employed in this study was primarily based on the classic method described by Kereszt et al. (2007) [59] and was further refined by incorporating the optimized conditions for soybean reported by Guo et al. (2011) [60]. The overexpression plasmid GmJAZ5-pBWA(V)BS and the corresponding empty vector pBWA(V)BS were transformed separately into competent cells of Agrobacterium rhizogenes strain K599. Transformants were selected on YEP solid medium supplemented with kanamycin (50 μg·mL^−1^) and streptomycin (50 μg·mL^−1^) at 28 °C. Positive colonies were subsequently verified by colony PCR. For inoculum preparation, 150 μL of the verified positive overnight culture was evenly spread onto fresh YEP solid medium containing the same antibiotics and incubated at 28 °C for 2 days to obtain fresh bacterial lawns. Prior to inoculation, bacterial cells were scraped directly from the plate surface using a sterile needle. Surface-sterilized and germinated ‘YC03-3’ seeds were sown in sterilized quartz sand. When the seedlings had fully expanded cotyledons and reached a height of approximately 7–10 cm (about 5 days after sowing), hypocotyl inoculation was performed. A sterile needle carrying fresh bacterial cells was used to puncture the hypocotyl approximately 0.5–1.0 cm below the cotyledonary node. The wound was immediately coated with additional bacterial cells to maximize contact between the bacteria and plant cells.

The inoculation site was maintained in a continuously moist state for 7 days to promote T-DNA transfer. Following inoculation, seedlings were co-cultivated under high humidity conditions (relative humidity > 90%) for 5 days. They were then transferred to a hydroponic system containing nutrient solution. The growth conditions were set as follows: temperature at 30 °C, photosynthetically active radiation at 400 μmol·m^−2^·s^−1^, relative humidity between 70% and 85%, and a photoperiod of 8 h of light followed by 16 h of darkness. The nutrient solution was renewed every 7 days. Approximately 14 days after inoculation, when the hairy roots emerging from the inoculation sites had sufficiently elongated, the original primary root was excised. Only the transgenic hairy roots originating from the inoculation wound were retained as the functional root system of the composite plants [60].

A total of 200 soybean seedlings were infected (100 each in the VC and OX groups). The number of plants in which the root hair system was successfully induced at the infection site was 90 in the VC group and 88 in the OX group (induction rates of 90% and 88%, respectively). Using the herbicide-resistance gene *bar* (bialaphos resistance) on the vector backbone as a selection marker, root hair systems from each composite plant were subjected to *bar* gene-specific PCR analysis (primers are provided in Supplementary Table S1), yielding 79 positive transgenic events in the VC group (positive rate 87.8%) and 75 in the OX group (positive rate 85.2%). *GmJAZ5* expression levels were further assessed by qRT-PCR, and independent events exhibiting expression levels at least five-fold higher than the VC control were selected for subsequent physiological analysis. Ultimately, 15 independent transgenic events from the VC group and 15 from the OX group were included in the analysis (each group comprised three biological replicates, with each biological replicate consisting of a mixture of root systems from five independent events). PCR gel electrophoresis images for transgenic identification and qRT-PCR expression data are shown in Supplementary Figure S1 and Table S2.

### 4.6. Determination of Reactive Oxygen Species (ROS) Levels

To directly verify the antioxidant effect of *GmJAZ5*, this study quantitatively determined the levels of superoxide anion (O_2_·^−^) and hydrogen peroxide (H_2_O_2_). The superoxide anion (O_2_·^−^) level was determined using the hydroxylamine oxidation method [61]. Fresh soybean root or leaf samples (0.5 g) were weighed, homogenized in an ice bath with 5 mL of pre-chilled 50 mM phosphate buffer (pH 7.8), and centrifuged at 12,000× *g* (equivalent to approximately 11,000 rpm, rotor model Beckman JA-25.50 (Beckman Coulter Commercial Enterprise (China) Co., Ltd., Shanghai, China)) at 4 °C for 15 min. The supernatant was collected for analysis. The reaction system consisted of 1 mL supernatant, 1 mL 50 mM phosphate buffer (pH 7.8), and 1 mL 1 mM hydroxylamine hydrochloride. After incubation at 25 °C for 1 h, 1 mL of 17 mM p-aminobenzenesulphonic acid and 1 mL of 7 mM α-naphthylamine were added, and the reaction was continued for a further 20 min at 25 °C. Absorbance was measured at 530 nm, and the rate of O_2_·^−^ formation was calculated from a NaNO_2_ standard curve and expressed as nmol·min^−1^·g^−1^ FW.

The hydrogen peroxide (H_2_O_2_) content was determined using the potassium iodide colorimetric method [62]. Fresh samples (0.5 g) were weighed, homogenized in an ice bath with 5 mL of pre-chilled 0.1% (*w*/*v*) trichloroacetic acid (TCA), and centrifuged at 12,000× *g* for 15 min at 4 °C. A 0.5 mL aliquot of the supernatant was mixed with 0.5 mL of 10 mM phosphate buffer (pH 7.0) and 1 mL of 1 M potassium iodide solution. After incubation in the dark for 1 h, absorbance was measured at 390 nm. A standard curve was prepared using a standard H_2_O_2_ solution (0–100 μM), and results were expressed as μmol·g^−1^ FW.

All ROS measurements were performed in quadruplicate, with each replicate being extracted and analyzed independently.

### 4.7. Determination of Antioxidant Enzyme Activities

Leaf and root samples (0.5 g each) from different salt concentration treatments were weighed and placed in a pre-chilled mortar. A total of 10 mL of pre-chilled 50 mM phosphate buffer (pH 7.8) was added in approximately three separate additions (approximately 3.3 mL each). The tissue was ground on ice until a homogeneous suspension was obtained, after which the suspension was transferred to a centrifuge tube and centrifuged at 4 °C and 10,000× *g* (equivalent to approximately 9500 rpm, rotor model Eppendorf F-45-30-11) for 20 min. The resulting supernatant was used as the enzyme extract.

Peroxidase (POD) activity was measured using the guaiacol method [63]. An aliquot of 40 μL of the enzyme extract was pipetted into a cuvette, and 3 mL of reaction mixture was added (containing 50 mM phosphate buffer, pH 6.0; final concentration of guaiacol approximately 2.5 mM; final concentration of H_2_O_2_ approximately 1.9 mM; prepared by adding 28 μL of guaiacol stock solution and 19 μL of 30% H_2_O_2_ to 50 mL of phosphate buffer, pH 6.0). The instrument was zeroed using phosphate buffer as a blank, and the change in OD_470_ was recorded every 30 s for a total of four readings.

Superoxide dismutase (SOD) activity was determined by the nitroblue tetrazolium (NBT) method [61]. A reaction mixture (2.9 mL; containing 50 mM phosphate buffer, pH 7.8, 14.5 mM methionine, 3 μM EDTA-Na_2_, 60 μM riboflavin and 2.25 μM NBT) and 0.1 mL of enzyme solution were added to a stoppered glass test tube. Two control tubes were prepared separately, each containing 2.9 mL of the reaction mixture and 0.1 mL of phosphate buffer; one tube was left uncovered as the maximum light-reduction control, whilst the other was shielded from light for zero calibration. All tubes were placed under 4000 lux illumination at 25 °C for 20 min, after which the OD_560_ of each tube was measured.

Catalase (CAT) activity was measured using a spectrophotometer (UV-5100B, Yuan Wei, Shanghai, China) [64]. The reaction solution (2.9 mL; containing 50 mM phosphate buffer, pH 7.0, and approximately 5.0 mM H_2_O_2_; prepared by adding 0.05 mL of 30% H_2_O_2_ to 100 mL of PBS, pH 7.0) was mixed with 0.1 mL of enzyme solution. The instrument was zeroed with phosphate buffer, and the change in absorbance at 240 nm was recorded every 30 s for a total of four readings.

Ascorbate peroxidase (APX) activity was measured according to the procedure described by Wang et al. [65]. Briefly, 0.10 mL of enzyme solution was added to 2.60 mL of reaction buffer (50 mM phosphate buffer, pH 7.0, 0.1 mM EDTA-Na_2_), followed by 0.15 mL of 5 mM AsA stock solution (final concentration approximately 0.25 mM) and 0.15 mL of 20 mM H_2_O_2_ stock solution (final concentration approximately 1.0 mM). The change in absorbance at 290 nm was recorded every 30 s for a total of four measurements.

All enzyme activity assays were performed with four biological replicates.

### 4.8. Determination of Soluble Protein and Thiobarbituric Acid-Reactive Substances (TBARS)

Soluble protein content was measured by the Coomassie Brilliant Blue staining (CBBS) method [66]. Briefly, 1 mL of enzyme solution was mixed with 5 mL of Coomassie Brilliant Blue solution, shaken thoroughly, and the absorbance at 595 nm was recorded after a 2 min reaction.

Malondialdehyde (MDA) is traditionally determined by the thiobarbituric acid (TBA) method; however, this method detects thiobarbituric acid-reactive substances (TBARS) rather than MDA per se, because compounds such as phenols and carbohydrates in plant tissues can also react with TBA, compromising detection specificity [67]. In this study, the results are expressed as “TBARS content” and serve as an indirect indicator of membrane lipid peroxidation.

TBARS content was determined using the TBA method as follows: 1 mL of enzyme solution was pipetted into a centrifuge tube, 2 mL of 0.6% TBA was added, and the mixture was thoroughly mixed. The tube was incubated in a boiling water bath at 100 °C for 15 min, rapidly cooled, and centrifuged at 4000× *g* (equivalent to approximately 6000 rpm, rotor model Eppendorf F-45-30-11 (Eppendorf China, Shanghai, China)) at room temperature for 20 min. A 3 mL aliquot of the supernatant was collected, and the absorbance values at 450 nm, 532 nm, and 600 nm were measured to calculate the TBARS content.

### 4.9. Determination of Ion Contents

Samples were dried in an oven at 80 °C for 48 h and subsequently ground into a fine powder. Approximately 0.1 g of the dried sample was digested in 5 mL of concentrated HNO_3_ (65%) at a temperature of 150 °C for 6 h [68]. After cooling, the digest was diluted to 25 mL with ultrapure water and filtered through a 0.45 μm membrane filter. Ion concentrations were measured using a flame atomic absorption spectrometer (AA7000, Shimadzu, Kyoto, Japan) at wavelengths of 589.0 nm for Na^+^ and 766.5 nm for K^+^. Standard curves were established using NaCl and KCl standard solutions ranging from 0 to 100 mg·L^−1^.

### 4.10. Determination of Hormone Contents

Endogenous levels of jasmonic acid (JA) and abscisic acid (ABA) were quantified using high-performance liquid chromatography (HPLC). For ABA analysis, a suitable amount of the sample was ground and extracted with 1 mL of pre-cooled 75% methanol (pH 3.5) at 4 °C overnight. Following centrifugation at 12,000× *g* (equivalent to approximately 13,000 rpm, rotor model Eppendorf F-45-30-11) for 15 min, the supernatant was evaporated to dryness under nitrogen and subsequently reconstituted in 0.3 mL of methanol. In the case of JA determination, a suitable amount of the sample was combined with 0.5 mL of a 60% methanol–acetic acid aqueous solution. This mixture was ground in liquid nitrogen and subjected to ultrasonic extraction for 20 min. After centrifugation at 12,000 rpm for 10 min, the supernatant was collected. The pellet underwent two additional extractions with the same solution, and the resulting supernatants were combined, evaporated to dryness under nitrogen, and reconstituted in 0.25 mL of pure methanol. Prior to analysis, both hormone samples were filtered through 0.22 μm syringe filters.

Chromatographic separation was conducted using a C18 column (250 mm × 4.6 mm, 5 μm) maintained at a temperature of 30 °C, with an injection volume of 10 μL. For the detection of abscisic acid (ABA), the mobile phase comprised methanol and a 1% acetic acid aqueous solution in a ratio of 40:60 (*v*/*v*), flowing at a rate of 0.8 mL·min^−1^, with detection occurring at 254 nm. In contrast, for jasmonic acid (JA) detection, the mobile phase included a mixture of methanol and acetonitrile (1:1, *v*/*v*) along with a 0.01% phosphoric acid aqueous solution in a ratio of 10:90 (*v*/*v*) at a flow rate of 1.0 mL·min^−1^, with detection at 210 nm [69].

### 4.11. Statistical Analysis

For physiological parameters, antioxidant enzyme activities, TBARS content, and soluble protein content were determined using four independent biological replicates (*n* = 4); ROS (O_2_·^−^ and H_2_O_2_) levels were measured using four biological replicates (*n* = 4); and the remaining parameters (ion concentrations, hormone concentrations, and gene expression) were measured using three independent biological replicates (*n* = 3). Each biological replicate consisted of a pooled sample from three independent transgenic events in order to minimize the influence of positional effects arising from individual transformation events.

For statistical analysis, the Shapiro–Wilk test was first used to assess data normality, followed by Levene’s test to assess homogeneity of variances. For data satisfying the assumptions of normal distribution and homogeneity of variances, comparisons among multiple groups were performed using one-way analysis of variance (ANOVA), with post hoc comparisons conducted using Duncan’s multiple range test (*p* < 0.05); pairwise comparisons between VC and OX were performed using Student’s *t*-test (two-tailed). For data that did not meet the assumptions of parametric tests, the Kruskal–Wallis non-parametric test was used, supplemented by Dunn’s post hoc test. All P-values were corrected for multiple comparisons using the Bonferroni method. Different lowercase letters indicate significant differences between groups (*p* < 0.05); * *p* < 0.05, ** *p* < 0.01, *** *p* < 0.001. Statistical analyses were performed using IBM SPSS Statistics 26.0, and figures and graphs were generated using GraphPad Prism 9.0 and Origin 2024.

## 5. Conclusions

In this study, *GmJAZ5* was overexpressed in soybean via Agrobacterium rhizogenes-mediated hairy root transformation. Overexpression significantly enhanced salt tolerance, as reflected by improved root growth, elevated activities of antioxidant enzymes (SOD, POD, APX, and CAT) and reduced MDA accumulation. *GmJAZ5* exhibited organ-specific expression under salt stress, being consistently suppressed in leaves but showing a biphasic pattern (initial suppression followed by upregulation) in roots. Physiological evidence further suggested that GmJAZ5 may contribute to salt tolerance by restricting Na^+^ transport to leaves and lowering the Na^+^/K^+^ ratio, accompanied by organ-specific modulation of JA and ABA levels. Together, these findings indicate that *GmJAZ5* plays an important role in coordinating soybean salt-stress responses, likely through crosstalk with multiple hormone signaling pathways. Further molecular studies using stable transgenic lines are warranted to elucidate the underlying regulatory mechanisms.

## Figures and Tables

**Table 1:** Effects of *GmJAZ5* overexpression on the phenotype of soybean composite plants.

	Treatment			
	0-VC	0-OX	100-VC	100-OX
Shoot Height (cm)	43.57 ± 3.53 b	49.90 ± 1.95 a	29.43 ± 2.25 d	34.40 ± 1.47 c
Root Length (cm)	22.67 ± 0.93 a	24.20 ± 1.45 a	16.10 ± 0.75 c	19.60 ± 0.53 b
Shoot Fresh Weight (g)	9.31 ± 0.60 b	12.14 ± 0.55 a	5.99 ± 0.47 d	7.68 ± 0.36 c
Root Fresh Weight (g)	2.23 ± 0.13 b	3.15 ± 0.20 a	1.12 ± 0.08 d	1.58 ± 0.03 c
Leaf Area (cm^2^)	166.23 ± 1.89 b	175.15 ± 7.77 a	143.92 ± 5.60 d	154.95 ± 1.36 c

Data are presented as mean ± standard deviation (SD) of three independent biological replicates, each comprising at least four plants. Different lowercase letters indicate significant differences among treatments (one-way ANOVA followed by Duncan’s multiple comparison test, *p* < 0.05).

## Data Availability

The original contributions presented in the study are publicly available.

## Supplementary Materials

The following supporting information can be downloaded at https://www.mdpi.com/article/10.3390/ijms27177829/s1.

## References

[1] Kim W.J., Kang B.H., Moon C.Y., Kang S., Shin S., Chowdhury S., Choi M.-S., Park S.-K., Moon J.-K., Ha B.-K. (2023). Quantitative Trait Loci (QTL) Analysis of Seed Protein and Oil Content in Wild Soybean (*Glycine soja*). Int. J. Mol. Sci..

[2] Yu X., Ben-Gal A., Scudiero E., Kisekka I., Rengasamy P., Yao R. (2025). Soil and Water Management to Prevent Salinization under Changing Climate Conditions. Agric. Water Manag..

[3] Zhang J. (2014). Salt-Affected Soil Resources in China. Coastal Saline Soil Rehabilitation and Utilization Based on Forestry Approaches in China.

[4] Munns R., Tester M. (2008). Mechanisms of Salinity Tolerance. Annu. Rev. Plant Biol..

[5] Zhu J.-K. (2016). Abiotic Stress Signaling and Responses in Plants. Cell.

[6] Wang W., Vinocur B., Altman A. (2003). Plant Responses to Drought, Salinity and Extreme Temperatures: Towards Genetic Engineering for Stress Tolerance. Planta.

[7] Achard P., Cheng H., De Grauwe L., Decat J., Schoutteten H., Moritz T., Van Der Straeten D., Peng J., Harberd N.P. (2006). Integration of Plant Responses to Environmentally Activated Phytohormonal Signals. Science.

[8] Ma L., Li J., Li J., Huo Y., Yang Y., Jiang C., Guo Y. (2026). Plant Salt-Tolerance Mechanisms: Classic Signaling Pathways, Emerging Frontiers, and Future Perspectives. Mol. Plant.

[9] Munns R. (2005). Genes and Salt Tolerance: Bringing Them Together. New Phytol..

[10] García-Caparrós P., Hasanuzzaman M., Lao M.T., Hasanuzzaman M., Fujita M., Oku H., Nahar K., Hawrylak-Nowak B. (2018). Ion Homeostasis and Antioxidant Defense Toward Salt Tolerance in Plants. Plant Nutrients and Abiotic Stress Tolerance.

[11] Shoukat E., Ahmed M.Z., Abideen Z., Azeem M., Ibrahim M., Gul B., Khan M.A. (2020). Short and Long Term Salinity Induced Differences in Growth and Tissue Specific Ion Regulation of *Phragmites karka*. Flora.

[12] Yang G., Sun R., Zhang Y., Song J., Li J., Luan Z., Qi W. (2026). Saline-Alkaline Stress Suppresses Soybean Germination and Early Seedling Growth via Induction of DNA Damage in Roots. Plants.

[13] Kazan K. (2015). Diverse Roles of Jasmonates and Ethylene in Abiotic Stress Tolerance. Trends Plant Sci..

[14] Wang X., Li Y., Chen Z., Li L., Li Q., Geng Z., Liu W., Hou R., Zhang L., Han D. (2025). *MbWRKY50* Confers Cold and Drought Tolerance through Upregulating Antioxidant Capacity Associated with ROS Scavenging. J. Plant Physiol..

[15] Han D., Zhang Z., Ding H., Wang Y., Liu W., Li H., Yang G. (2018). Molecular Cloning and Functional Analysis of *MbWRKY3* Involved in Improved Drought Tolerance in Transformed Tobacco. J. Plant Interact..

[16] Li W., Li H., Wei Y., Han J., Wang Y., Li X., Zhang L., Han D. (2024). Overexpression of a *Fragaria vesca* NAM, ATAF, and CUC (NAC) Transcription Factor Gene (*FvNAC29*) Increases Salt and Cold Tolerance in *Arabidopsis thaliana*. Int. J. Mol. Sci..

[17] Han J., Dai J., Chen Z., Li W., Li X., Zhang L., Yao A., Zhang B., Han D. (2024). Overexpression of a ‘Beta’ MYB Factor Gene, *VhMYB15*, Increases Salinity and Drought Tolerance in *Arabidopsis thaliana*. Int. J. Mol. Sci..

[18] Ashraf M., Wu L. (1994). Breeding for Salinity Tolerance in Plants. Crit. Rev. Plant Sci..

[19] Ghorbel M., Brini F., Sharma A., Landi M. (2021). Role of Jasmonic Acid in Plants: The Molecular Point of View. Plant Cell Rep..

[20] Sheteiwy M.S., Ulhassan Z., Qi W., Lu H., AbdElgawad H., Minkina T., Sushkova S., Rajput V.D., El-Keblawy A., Jośko I. (2022). Association of Jasmonic Acid Priming with Multiple Defense Mechanisms in Wheat Plants under High Salt Stress. Front. Plant Sci..

[21] Wang J., Song L., Gong X., Xu J., Li M. (2020). Functions of Jasmonic Acid in Plant Regulation and Response to Abiotic Stress. Int. J. Mol. Sci..

[22] Prerostova S., Dobrev P.I., Gaudinova A., Knirsch V., Körber N., Pieruschka R., Fiorani F., Motyka V., Vankova R. (2017). Hormonal Dynamics during Salt Stress Responses of Salt-Sensitive Arabidopsis thaliana and Salt-Tolerant Thellungiella salsuginea. Plant Sci..

[23] De Domenico S., Taurino M., Gallo A., Poltronieri P., Pastor V., Flors V., Santino A. (2019). Oxylipin Dynamics in Medicago truncatula in Response to Salt and Wounding Stresses. Physiol. Plant..

[24] Gu D., Liu X., Wang M., Zheng J., Hou W., Wang G., Wang J. (2008). Overexpression of *ZmOPR1* in Arabidopsis Enhanced the Tolerance to Osmotic and Salt Stress during Seed Germination. Plant Sci..

[25] Fang L.C., Su L.Y., Sun X.M., Li X.B., Sun M.X., Karungo S.K., Fang S., Chu J.F., Li S.H., Xin H.P. (2016). Expression of *Vitis amurensis* NAC26 in *Arabidopsis* Enhances Drought Tolerance by Modulating Jasmonic Acid Synthesis. J. Exp. Bot..

[26] Zhao X., He Y., Liu Y., Wang Z., Zhao J. (2024). JAZ Proteins: Key Regulators of Plant Growth and Stress Response. Crop J..

[27] Chini A., Gimenez-Ibanez S., Goossens A., Solano R. (2016). Redundancy and Specificity in Jasmonate Signalling. Curr. Opin. Plant Biol..

[28] Pauwels L., Barbero G.F., Geerinck J., Tilleman S., Grunewald W., Pérez A.C., Chico J.M., Bossche R.V., Sewell J., Gil E. (2010). NINJA Connects the Co-Repressor TOPLESS to Jasmonate Signalling. Nature.

[29] Zhang F., Yao J., Ke J., Zhang L., Lam V.Q., Xin X.-F., Zhou X.E., Chen J., Brunzelle J., Griffin P.R. (2015). Structural Basis of JAZ Repression of MYC Transcription Factors in Jasmonate Signalling. Nature.

[30] Wei X., Liu Y., Liu Y., Yin X., Xie T., Chen R., Wei Q. (2021). Advances of JAZ Family in Plants. Plant Physiol. J..

[31] Zhang F., Ke J., Zhang L., Chen R., Sugimoto K., Howe G.A., Xu H.E., Zhou M., He S.Y., Melcher K. (2017). Structural Insights into Alternative Splicing-Mediated Desensitization of Jasmonate Signaling. Proc. Natl. Acad. Sci. USA.

[32] Campos M.L., Yoshida Y., Major I.T., de Oliveira Ferreira D., Weraduwage S.M., Froehlich J.E., Johnson B.F., Kramer D.M., Jander G., Sharkey T.D. (2016). Rewiring of Jasmonate and Phytochrome B Signalling Uncouples Plant Growth-Defense Tradeoffs. Nat. Commun..

[33] Yao D., Zhang X., Zhao X., Liu C., Wang C., Zhang Z., Zhang C., Wei Q., Wang Q., Yan H. (2011). Transcriptome Analysis Reveals Salt-Stress-Regulated Biological Processes and Key Pathways in Roots of Cotton (*Gossypium hirsutum* L.). Genomics.

[34] Zhu D., Cai H., Luo X., Bai X., Deyholos M.K., Chen Q., Chen C., Ji W., Zhu Y. (2012). Over-Expression of a Novel JAZ Family Gene from *Glycine soja*, Increases Salt and Alkali Stress Tolerance. Biochem. Biophys. Res. Commun..

[35] Yamada S., Kano A., Tamaoki D., Miyamoto A., Shishido H., Miyoshi S., Taniguchi S., Akimitsu K., Gomi K. (2012). Involvement of *OsJAZ8* in Jasmonate-Induced Resistance to Bacterial Blight in Rice. Plant Cell Physiol..

[36] Ye H., Du H., Tang N., Li X., Xiong L. (2009). Identification and Expression Profiling Analysis of TIFY Family Genes Involved in Stress and Phytohormone Responses in Rice. Plant Mol. Biol..

[37] Valenzuela C.E., Acevedo-Acevedo O., Miranda G.S., Vergara-Barros P., Holuigue L., Figueroa C.R., Figueroa P.M. (2016). Salt Stress Response Triggers Activation of the Jasmonate Signaling Pathway Leading to Inhibition of Cell Elongation in *Arabidopsis* Primary Root. J. Exp. Bot..

[38] Niu M., Bie Z., Huang Y. (2022). Root-Borne Signals and Their Control of Guard Cell Operation under Saline Conditions: The Role of Root Signals in Stomata Regulation. Advances in Botanical Research.

[39] Chini A., Fonseca S., Fernández G., Adie B., Chico J.M., Lorenzo O., García-Casado G., López-Vidriero I., Lozano F.M., Ponce M.R. (2007). The JAZ Family of Repressors Is the Missing Link in Jasmonate Signalling. Nature.

[40] Withers J., Yao J., Mecey C., Howe G.A., Melotto M., He S.Y. (2012). Transcription Factor-Dependent Nuclear Localization of a Transcriptional Repressor in Jasmonate Hormone Signaling. Proc. Natl. Acad. Sci. USA.

[41] Jin J., Wang J., Li K., Wang S., Qin J., Zhang G., Na X., Wang X., Bi Y. (2021). Integrated Physiological, Transcriptomic, and Metabolomic Analyses Revealed Molecular Mechanism for Salt Resistance in Soybean Roots. Int. J. Mol. Sci..

[42] Fujita M., Hasanuzzaman M. (2022). Approaches to Enhancing Antioxidant Defense in Plants. Antioxidants.

[43] Foyer C.H., Noctor G. (2011). Ascorbate and Glutathione: The Heart of the Redox Hub. Plant Physiol..

[44] Kang D.-J., Seo Y.-J., Lee J.-D., Ishii R., Kim K.U., Shin D.H., Park S.K., Jang S.W., Lee I.-J. (2005). Jasmonic Acid Differentially Affects Growth, Ion Uptake and Abscisic Acid Concentration in Salt-Tolerant and Salt-Sensitive Rice Cultivars. J. Agron. Crop Sci..

[45] Sirhindi G., Mir M.A., Abd-Allah E.F., Ahmad P., Gucel S. (2016). Jasmonic Acid Modulates the Physio-Biochemical Attributes, Antioxidant Enzyme Activity, and Gene Expression in *Glycine max* under Nickel Toxicity. Front. Plant Sci..

[46] Ayala A., Muñoz M.F., Argüelles S. (2014). Lipid Peroxidation: Production, Metabolism, and Signaling Mechanisms of Malondialdehyde and 4-Hydroxy-2-Nonenal. Oxid. Med. Cell. Longev..

[47] Hasanuzzaman M., Raihan M.R.H., Masud A.A.C., Rahman K., Nowroz F., Rahman M., Nahar K., Fujita M. (2021). Regulation of Reactive Oxygen Species and Antioxidant Defense in Plants under Salinity. Int. J. Mol. Sci..

[48] Guo H.J., Huang Z.J., Li M.Q., Hou Z.N. (2020). Growth, Ionic Homeostasis, and Physiological Responses of Cotton under Different Salt and Alkali Stresses. Sci. Rep..

[49] Cao D., Hou W., Liu W., Yao W., Wu C., Liu X., Han T. (2011). Overexpression of *TaNHX2* Enhances Salt Tolerance of ‘Composite’ and Whole Transgenic Soybean Plants. Plant Cell Tissue Organ Cult..

[50] Yu Z., Duan X., Luo L., Dai S., Ding Z., Xia G. (2020). How Plant Hormones Mediate Salt Stress Responses. Trends Plant Sci..

[51] Wasternack C., Hause B. (2013). Jasmonates: Biosynthesis, Perception, Signal Transduction and Action in Plant Stress Response, Growth and Development: An Update to the 2007 Review in *Annals of Botany*. Ann. Bot..

[52] Schulze A., Zimmer M., Mielke S., Stellmach H., Melnyk C.W., Hause B., Gasperini D. (2019). Wound-Induced Shoot-to-Root Relocation of JA-Ile Precursors Coordinates *Arabidopsis* Growth. Mol. Plant.

[53] Zhu T., Herrfurth C., Xin M., Savchenko T., Feussner I., Goossens A., De Smet I. (2021). Warm Temperature Triggers JOX- and ST2A-Mediated Jasmonate Catabolism to Promote Plant Growth. Nat. Commun..

[54] Shu K., Qi Y., Chen F., Meng Y., Luo X., Shuai H., Zhou W., Ding J., Du J., Liu J. (2017). Salt Stress Represses Soybean Seed Germination by Negatively Regulating GA Biosynthesis while Positively Mediating ABA Biosynthesis. Front. Plant Sci..

[55] Liang C., Piñeros M.A., Tian J., Yao Z., Sun L., Liu J., Shaff J., Coluccio A., Kochian L.V., Liao H. (2013). Low pH, Aluminum, and Phosphorus Coordinately Regulate Malate Exudation through *GmALMT1* to Improve Soybean Adaptation to Acid Soils. Plant Physiol..

[56] Xue Y.B., Zhuang Q.L., Zhu S.N., Xiao B.X., Liang C.Y., Liao H., Tian J. (2018). Genome-Wide Transcriptome Analysis Reveals Complex Regulatory Mechanisms Underlying Phosphate Homeostasis in Soybean Nodules. Int. J. Mol. Sci..

[57] Vo T.T.B., Tabassum M., Nattanong B., Im H.J., Kim M., Pan L.L., Byun H.S., Kwak H.R., Lee S. (2026). Reverse Genetics Platform for Tomato Mottle Mosaic Virus: Infectious Clone Construction and Resistance Screening in Tomato. Mol. Biol. Rep..

[58] Liu P.-D., Xue Y.-B., Chen Z.-J., Liu G.-D., Tian J. (2016). Characterization of Purple Acid Phosphatases Involved in Extracellular dNTP Utilization in *Stylosanthes*. J. Exp. Bot..

[59] Kereszt A., Li D., Indrasumunar A., Nguyen C.D.T., Nontachaiyapoom S., Kinkema M., Gresshoff P.M. (2007). *Agrobacterium rhizogenes*-Mediated Transformation of Soybean to Study Root Biology. Nat. Protoc..

[60] Guo W., Zhao J., Li X., Qin L., Yan X., Liao H. (2011). A Soybean β-Expansin Gene *GmEXPB2* Intrinsically Involved in Root System Architecture Responses to Abiotic Stresses. Plant J..

[61] Dey S., Raychaudhuri S.S. (2024). Methyl Jasmonate Improves Selenium Tolerance via Regulating ROS Signalling, Hormonal Crosstalk and Phenylpropanoid Pathway in *Plantago ovata*. Plant Physiol. Biochem..

[62] Velikova V., Yordanov I., Edreva A. (2000). Oxidative Stress and Some Antioxidant Systems in Acid Rain-Treated Bean Plants: Protective Role of Exogenous Polyamines. Plant Sci..

[63] Rácz A., Hideg É., Czégény G. (2018). Selective Responses of Class III Plant Peroxidase Isoforms to Environmentally Relevant UV-B Doses. J. Plant Physiol..

[64] Srivastava A.K., Singh D. (2020). Assessment of Malathion Toxicity on Cytophysiological Activity, DNA Damage and Antioxidant Enzymes in Root of *Allium cepa* Model. Sci. Rep..

[65] Wang X., Wu Z., Zhou Q., Wang X., Song S., Dong S. (2022). Physiological Response of Soybean Plants to Water Deficit. Front. Plant Sci..

[66] Ren H., Zhang B., Zhang F., Liu X., Wang X., Zhang C., Zhao K., Yuan R., Lamlom S.F., Abdelghany A.M. (2024). Integration of Physiological and Transcriptomic Approaches in Investigating Salt-Alkali Stress Resilience in Soybean. Plant Stress.

[67] Johnston C.W., Wagner U., Latowski D., Kruk J., Strzalka K. (2007). The Oxidative Degradation of Chlorophyll in the Model System of Thylakoids: A New Methodology for Detecting Thiobarbituric Acid Reactive Substances. Plant Physiol. Biochem..

[68] Havlin J.L., Soltanpour P.N. (1980). A Nitric Acid Plant Tissue Digest Method for Use with Inductively Coupled Plasma Spectrometry. Commun. Soil Sci. Plant Anal..

[69] Wu M., Yi Y., Zhang C.Y., Liu J., Chang J., Cheng X., Zhang L. (2024). Application of ultra-performance liquid chromatography–tandem mass spectrometry for the simultaneous determination of three endogenous plant hormones in crops. Exp. Technol. Manag..

